# Epigenetically-driven anatomical diversity of synovial fibroblasts guides joint-specific fibroblast functions

**DOI:** 10.1038/ncomms14852

**Published:** 2017-03-23

**Authors:** Mojca Frank-Bertoncelj, Michelle Trenkmann, Kerstin Klein, Emmanuel Karouzakis, Hubert Rehrauer, Anna Bratus, Christoph Kolling, Maria Armaka, Andrew Filer, Beat A. Michel, Renate E. Gay, Christopher D. Buckley, George Kollias, Steffen Gay, Caroline Ospelt

**Affiliations:** 1 Center of Experimental Rheumatology, University Hospital Zurich and University of Zurich, Wagistrasse 14, 8952 Schlieren, Zurich, Switzerland; 2 Functional Genomics Center Zurich, ETH Zurich and University of Zurich, Winterthurerstrasse 190, 8057 Zurich, Switzerland; 3 Schulthess Clinic, Lengghalde 2, 8008 Zurich, Switzerland; 4 Division of Immunology, Biomedical Sciences Research Center ‘Alexander Fleming', 34, Fleming Street, 16672 Vari, Attica, Greece; 5 Institute of Inflammation and Ageing (IIA), University of Birmingham, Queen Elizabeth Hospital, Birmingham B15 2WB, UK; 6 Department of Experimental Physiology, School of Medicine, National and Kapodistrian University of Athens, 75 Mikras Asias Street, 11527 Goudi, Athens, Greece; 7 Center of Applied Biotechnology and Molecular Medicine (CABMM), University of Zurich, Winterthurerstrasse 190, 8057 Zurich, Switzerland

## Abstract

A number of human diseases, such as arthritis and atherosclerosis, include characteristic pathology in specific anatomical locations. Here we show transcriptomic differences in synovial fibroblasts from different joint locations and that HOX gene signatures reflect the joint-specific origins of mouse and human synovial fibroblasts and synovial tissues. Alongside DNA methylation and histone modifications, bromodomain and extra-terminal reader proteins regulate joint-specific HOX gene expression. Anatomical transcriptional diversity translates into joint-specific synovial fibroblast phenotypes with distinct adhesive, proliferative, chemotactic and matrix-degrading characteristics and differential responsiveness to TNF, creating a unique microenvironment in each joint. These findings indicate that local stroma might control positional disease patterns not only in arthritis but in any disease with a prominent stromal component.

## Abstract (web summary)

Arthritis affects different joints variably despite systemic inflammatory cues. Here the authors show anatomical differences in the transcriptome, epigenome and function of synovial fibroblasts that might affect susceptibility to site-specific joint diseases.

Formation of joint-specific structures during embryogenesis is accompanied by tightly controlled topographical and temporal expression of specific sets of positional genes, including homeobox (HOX) transcription factors^1^. The highly conserved HOX transcription factors specify regional identities of cells and tissues throughout the body and regulate the correct formation of the body axes^2^,^3^. Adult human cells and tissues, such as skin fibroblasts^4^,^5^,^6^, aortic smooth muscle cells^7^ and adipose tissue depots^8^, retain key features of embryonic positional HOX gene expression. The unique topographical signature of HOX transcription factors and downstream effector molecules in skin fibroblasts provides critical signals to guide the site-specific fate of neighbouring cells, such as keratinocytes^9^.

Most forms of arthritis, including rheumatoid arthritis (RA), osteoarthritis (OA) and spondyloarthropathies, occur with a distinctive topographical pattern of joint involvement^10^. RA is a symmetric, destructive polyarticular arthritis that is typically localized distally in small joints of the hands and feet, in particular metacarpophalangeal joints and metatarsophalangeal (MTP) joints. Proximal, larger joints, like knees and shoulders, are involved less often and usually later in disease. In spondyloarthropathies, on the other hand, involvement of the spine together with peripheral oligoarthritis, affecting primarily the joints of the lower extremity, is typical. OA, which is a chronic, primarily degenerative disease of the cartilage, is also found mainly in knees and hips. In addition, OA can affect the feet, typically the MTP I joint, and hands, where distal interphalangeal joints are primarily involved.

Arthritis-specific topographical patterns of joint involvement form despite common systemic activation of the adaptive immune system and common systemic and environmental risk factors, arguing for a local predisposing factor within the synovium. Resident stromal cells of the synovium have a central function in the pathogenesis of arthritis. Synovial fibroblasts (SFs), the most abundant resident stromal cells of the synovium, are major local effectors of joint inflammation and destruction in arthritis, producing a variety of proinflammatory and matrix-degrading molecules^11^,^12^,^13^. *In vivo* studies suggest that SFs from patients with RA can transmigrate into vasculature, spreading RA from oligoarticular to polyarticular disease^12^.

Here we demonstrate that SFs of different anatomical origins have different transcriptomes that translates into unique joint-specific phenotypes of SFs. This finding might underlie the joint-specific occurrence and severity of arthritis.

## Results

### SFs cluster according to joint of origin

**Figure 1:**
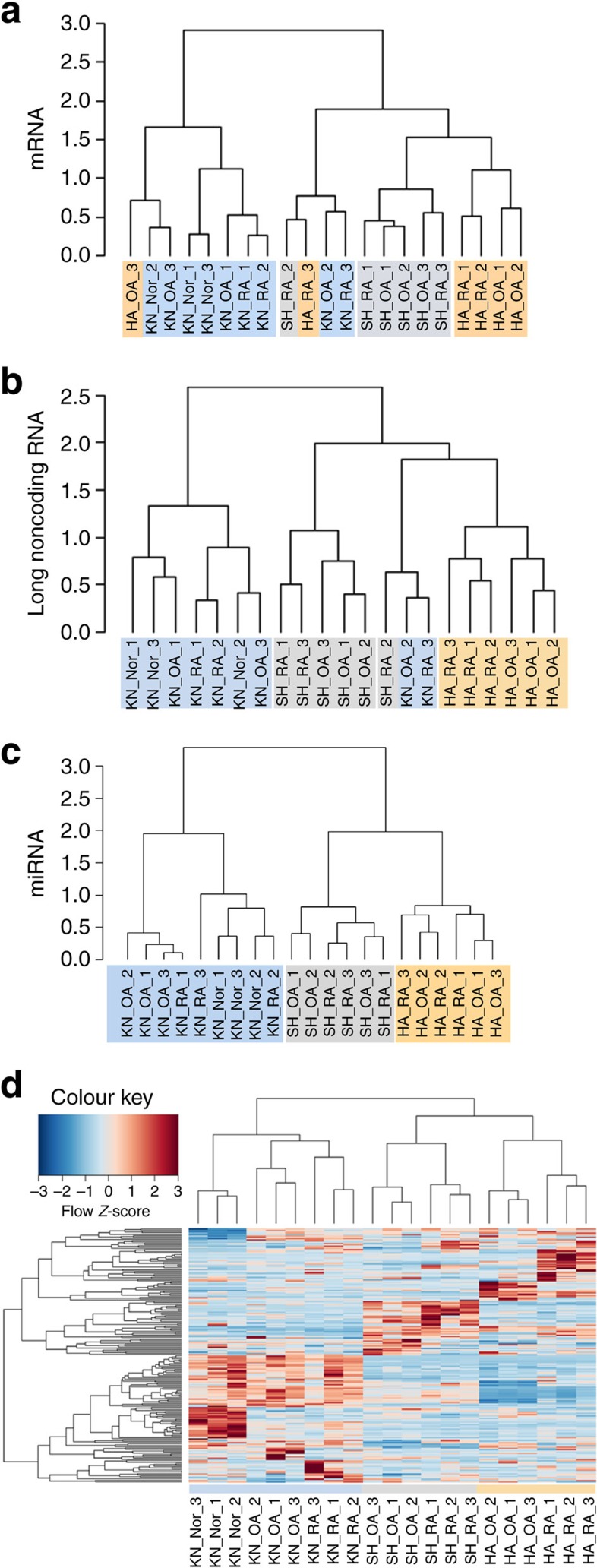
Cluster analysis of RNA sequencing in human SFs from different locations. Unsupervised hierarchical clustering of SFs (*n*=21) from knees (KN, blue), shoulders (SH, grey) and hands (HA, orange) of rheumatoid arthritis (RA) and osteoarthritis (OA) patients and knees of non-arthritic subjects (norm) (see Supplementary Table 1) based on RNA-seq data. The 100 genes (protein coding, long noncoding and microRNA) with the biggest variance across all samples were used. The cluster analysis is based on normalized reads after log transformation with an offset of 10 counts added: (**a**) mRNA—one hand OA sample clustered into the knee SF group, and one OA knee and one RA knee samples clustered with upper extremity joints; (**b**) long noncoding RNA—increased accuracy of topographical clustering of SF, one RA and one OA knee SF clustered into the upper extremity joint group; and (**c**) microRNA—accurate topographical clustering into shoulder, hand and knee SFs. (**d**) Supervised cluster analysis showing 196 long transcripts (see Supplementary Data 1) identified as the 25 most significant genes (satisfying a minimum log2 difference of 0.5 and maximum *P* value of 0.05) in pairwise comparisons between joint locations (knee, shoulder, hand) and diagnoses (RA, OA, norm). Heatmaps display *Z*-scores of normalized read counts.

Site-specific transcriptional programmes regulate regional diversity of skin^9^,^14^,^15^. Similar to skin, joints show remarkable anatomical diversity in structure, function and susceptibility to disease. To investigate the anatomical diversity of transcriptional programmes in SFs, we sequenced total RNA from cultured knee, shoulder and hand SFs from RA and OA patients and from knees of non-arthritic subjects with joint pain (see Supplementary Table 1 for patient characteristics). Joint locations were selected to span the proximal–distal (shoulder versus hand) and upper–lower (shoulder and hand versus knee) extremities in concordance with developmental limb axes^16^. Unsupervised hierarchical cluster analysis of RNA sequencing data showed that SFs cluster according to anatomical joint location rather than diagnosis (Fig. 1). When based on messenger RNA (mRNA) expression (Fig. 1a) the accuracy of positional clustering was comparable to what was reported for adult dermal fibroblasts^9^. The accuracy of the topographical clustering in SFs was improved when clustering was based on long noncoding RNA (lncRNA) (Fig. 1b) and a perfect clustering into shoulder, hand and knee SFs was achieved based on microRNA (Fig. 1c). We confirmed differential expression of six of the most significantly changed microRNAs in an additional set of SFs from hands, shoulders and knees with real-time PCR (Supplementary Fig. 1). Among the noncoding RNA, lncRNA *HOTAIR* and *HOTTIP* were the most differentially expressed transcripts between upper versus lower extremity SFs and distal versus proximal SFs, respectively. In addition, several transcripts with known roles in arthritis were positionally enriched in specific joint locations including *MMP1*, *MMP13*, *interleukin (IL) 1R*, *IL34* and *CXCL12* (refs 17, 18, 19, 20).

Next, we searched for genes that would explicitly distinguish between SFs from different anatomical locations and within each joint between different diagnoses using supervised cluster analysis (Fig. 1d). Our model of supervised clustering retrieved 196 transcripts (Supplementary Data 1) that were uniquely expressed in SFs at specific anatomical locations and within each location exclusively discriminated between SFs from distinct disease states. The identified genes included several factors involved in positional cell identity and limb patterning (*HOTAIR*, *HOXA11*, *HOXA13*, *HOTTIP*, *TBX5*, *TBX2*, *HAND2*, *MEIS1*, *MEIS2*), molecules implicated in wnt signalling (*SFRP4, ROR2*), chemotaxis and inflammation (*IL18*, *IL17D*, *CCL28*) and cell–cell interactions (*ITGA8*, *ITG2B*).

These data showed that clear topographical differences in gene expression patterns exist between SFs from joints of different anatomical locations. The noncoding transcriptome exhibited the most distinct differences between SFs from various joints.

### HOX genes define anatomical origin of SFs

**Figure 2:**
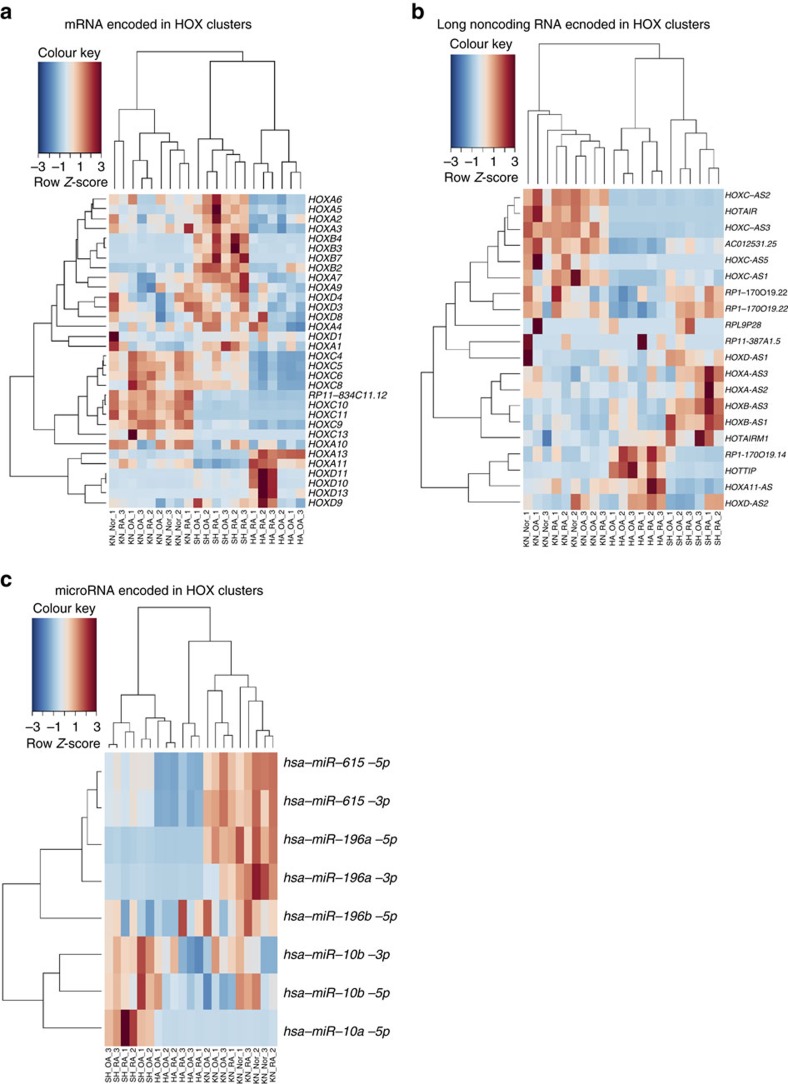
Cluster analysis based on HOX-encoded transcripts in human SFs from different locations. Cluster analysis of RNA sequencing data using (**a**) protein-coding RNA, (**b**) long noncoding RNA and (**c**) microRNA transcripts encoded in HOX loci clearly discriminated between SFs of different anatomical locations (*n*=21). Heatmaps display *Z*-scores of normalized read counts. Genes encoded within HOX loci were identified using the HOX gene annotation in GRCh38.p2, Ensembl Release 80 (May 2015). Only transcripts expressed above the threshold level were used in the cluster analysis. HA, hand; KN, knee; Nor, non-arthritic joint pain; OA, osteoarthritis; RA, rheumatoid arthritis; SH, shoulder.

Since a substantial number of the most differentially expressed transcripts between different joint locations were transcripts encoded in the HOX loci, we explored whether the transcripts in the HOX loci are sufficient to assign SF samples to the correct joint location. Cluster analysis of RNA sequencing data using exclusively transcripts encoded in HOX loci and expressed above the threshold level showed that protein-coding transcripts (Fig. 2a) as well as lncRNA (Fig. 2b) and microRNA (Fig. 2c) encoded in the HOX loci are sufficient to cluster SFs according to joint location. Transcripts encoded in the 5′ end of the HOXA (*HOXA11-AS*, *HOXA13*, *HOTTIP*) and HOXD (*HOXD10*, *HOXD11*, *HOXD13*) clusters reflected the positional identity to SFs from distal (hands) compared with more proximal (shoulder, knee) joints. Shoulder-specific expression in SFs was found in HOX transcripts encoded in the 3′ end of the HOXA, HOXB and HOXD clusters. Transcripts encoded in the 5′ end of HOXC locus (*miR-196a*, *HOXC-AS1*, *HOXC-AS2*, *HOXC-AS3*, *HOTAIR* and *HOXC13*) distinguished knee from upper extremity SFs. This HOX signature recapitulated key features of the embryonic positional HOX gene expression along the proximal–distal and anterior–posterior developmental axes^2^,^16^,^21^.

**Figure 3:**
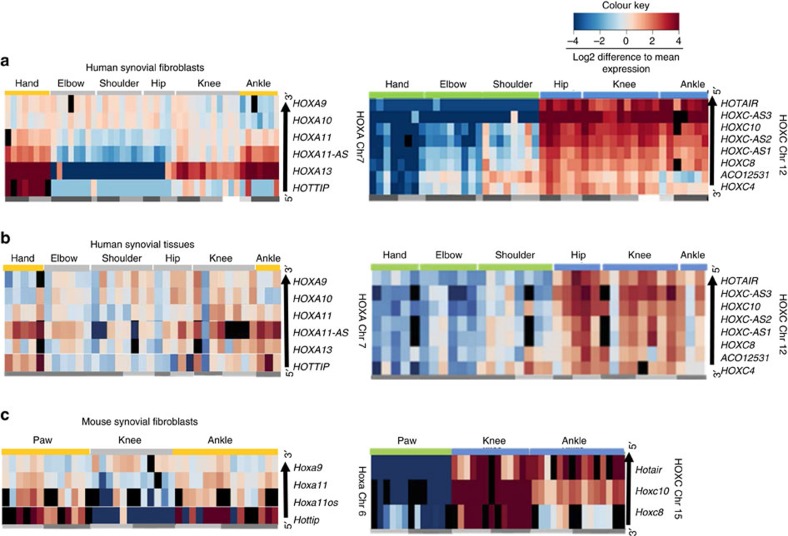
Conservation of positional HOX gene signatures. (**a**) Heatmaps based on the qPCR measurements of selected HOXA and HOXC transcripts in human SFs (see Supplementary Table 2) from hands (*n*=8), elbows (*n*=8), shoulders (*n*=8), hips (*n*=6), knees (*n*=9) and ankles (*n*=7): the 5′ end-encoded HOXA and HOXC transcripts are key factors in discriminating proximal versus distal and upper versus lower extremity SFs. (**b**) Heatmaps based on the qPCR measurements of selected HOXA and HOXC transcripts in human synovial tissues (see Supplementary Table 3) from hands (*n*=5), elbows (*n*=6), shoulders (*n*=8), hips (*n*=5), knees (*n*=8) and ankles (*n*=3), recapitulating the proximal–distal HOXA and the upper–lower extremity HOXC signatures of SFs. (**c**) Heatmaps based on the qPCR measurements of selected *HoxA* and *HoxC* transcripts in mouse SFs from front paws (*n*=13), knees (*n*=12) and ankles (*n*=15) of healthy wild-type and arthritic TNF transgenic mice (see Supplementary Table 4). Dark grey side lines: rheumatoid arthritis, TNF transgenic mice; light grey: osteoarthritis, wild-type mice; very light grey: resolving arthritis; white: non-arthritic subjects. The order of HOX transcripts in the heatmaps follows the 5′ to 3′ spatial organization of HOX loci (arrows next to heatmaps). Black squares are samples not measured. Heatmaps are based on the log2 ratios of sample ΔCT (CT target gene−CT endogenous control gene) expression values relative to the average ΔCT of a gene across all the samples as measured by qPCR.

Measuring selected HOX transcripts in cultured SFs from hands, elbows, shoulders, hips, knees and ankles (see Supplementary Table 2 for patient characteristics) by real-time PCR further confirmed the selective transcriptional activation of the 5′ end of the HOXA and HOXC clusters in SFs from distal (hands and ankles) and lower extremity joints, respectively (Fig. 3a, Supplementary Data 2). The lncRNA *HOTAIR*, which was reported to be expressed at posterior and distal anatomical sites^22^, was repressed in upper extremity joints and expressed in lower extremity joints. *HOTAIR* expression did not differ between hip, knee and ankle SFs, but SFs from proximal interphalangeal joints of the toe (*n*=1, Supplementary Fig. 2) expressed higher levels of *HOTAIR*. Moving along the HOXA and HOXC clusters in a 5′ to 3′ direction, the transcripts were expressed sequentially in SFs from proximal joints (hip, shoulder) and joints of the upper extremity, respectively. This corresponded to the developmental body axes and the spatial collinearity of HOX genes with their genomic 5′ to 3′ position in the cluster. Yet, the expression of *HOXA11*, which was demonstrated as central to the morphogenesis of mouse zeugopod (elbow), while largely dispensable for autopod (paw), was higher in hand (human autopod) compared with elbow (zeugopod) SFs.

### Positional HOX signature is maintained in synovial tissues

RA synovial tissues are characterized by a heterotrophic lining layer, neoangiogenesis and leukocyte infiltration and are highly heterogeneous with respect to synovial histomorphology and type of infiltrating leukocytes^23^. OA synovium can show signs of patchy synovitis of lower grade compared with RA synovium^24^. Despite cellular heterogeneity, the positional pattern of HOXA and HOXC gene expression of SFs was largely reproduced in synovial tissues (Fig. 3b and Supplementary Data 2) from different joints of RA and OA patients (see Supplementary Table 3 for patient characteristics). Given this, SFs appear the principal cell type conferring positional identity on the synovium; yet, the contribution of other cells, including endothelial^25^ and vascular smooth muscle cells^7^, remains to be explored. Maintenance of positional HOX signatures in the synovium also demonstrates that positional expression of HOX transcripts in SFs is not an *in vitro* artefact of cell culture conditions.

### HOX gene signatures are conserved in mouse SF

In concordance with human SFs, adult mouse SFs from wild-type and tumour necrosis factor (TNF) transgenic C57BL/6 mice (Supplementary Table 4) showed a strict positional expression pattern of the measured coding and noncoding HoxA and HoxC transcripts (Fig. 3c, Supplementary Fig. 3 and Supplementary Data 2). TNF transgenic mice spontaneously develop a chronic, destructive arthritis^26^. None of the measured positional 5′ HoxA and HoxC transcripts differed significantly between healthy wild-type and arthritic TNF transgenic mouse SFs, nor between RA and OA SFs or synovial tissues of the same anatomical location (Fig. 3 and Supplementary Data 2). This infers that the expression of these HOX transcripts is not arthritis dependent, but position specific. Similar to the topographical expression during embryogenesis^21^, location-specific expression of 5′-encoded HOXA and HOXC transcripts in adult SFs appeared to be mainly conserved across humans and mice. However, several lncRNA species, in particular in the HOXA locus, are not conserved between human and mice and might therefore confer human-specific functions. In addition, the expression of *miR-196a* encoded in HOXC did not follow the strict anterior–posterior pattern in mouse (Supplementary Fig. 3) as seen in human SFs (Supplementary Fig. 1).

Together, these data confirm that adult human and mouse SFs from different anatomical locations exhibit joint-specific HOXA and HOXC signatures that are maintained over several passages in cell culture conditions, are arthritis independent and reproduced in whole synovial tissues.

### Positional HOX gene expression is epigenetically regulated

**Figure 4:**
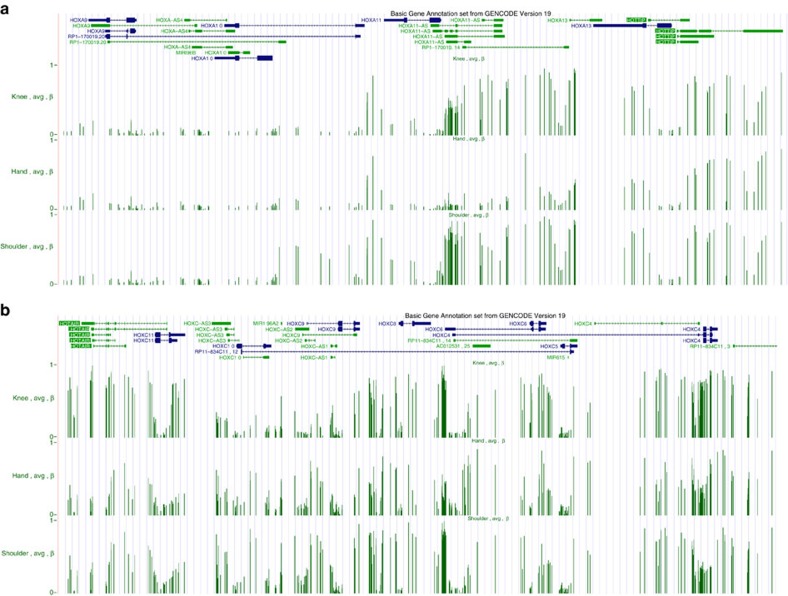
Joint-specific DNA methylation landscapes of the HOXA and HOXC clusters in synovial fibroblasts. Visualization of β-values of hand, knee and shoulder SFs in the UCSC genome browser showed (**a**) hypomethylation of the 5′ HOXA-encoded genes in hand versus knee or shoulder SFs and (**b**) hypomethylation of *HOTAIR* and *HOXC11* loci in hand and shoulder versus knee SF, while genes in the central part of the HOXC cluster mainly showed gene body hypermethylation in joints of the upper extremity.

It has been previously shown that the expression of positional HOX determinants during embryogenesis is stabilized via epigenetic mechanisms, including DNA methylation^27^, histone acetylation^28^ and histone methylation^28^. Our analysis of the HOXA and HOXC loci in SFs showed less methyl marks in the 5′ HOXA locus in hand compared with shoulder and knee SFs, corresponding to the exclusive expression of the transcripts from the 5′ end of the HOXA locus in hand SFs (Fig. 4a). In the HOXC locus, DNA methylation patterns at the 5′ tip and central part of the cluster (*HOTAIR* to *HOXC9*) discriminated knee from hand and shoulder SFs (posterior versus anterior body axis); however, the relationship between joint-specific DNA methylation patterns and positional gene expression was more complex than in the HOXA locus. DNA of the central part of the HOXC cluster (between *HOXC10* promoter and *HOXC9* locus) was hypermethylated in hands and shoulders versus knees (Fig. 4b), corresponding to transcriptional repression of these loci in upper extremity joints (Fig. 3a). In contrast, DNA at the 5′ tip of the HOXC cluster (*HOXC11*, *HOTAIR*) was hypomethylated in upper extremity joints, where the locus is transcriptionally silent, compared with knees, where the locus is actively transcribed. Indeed, DNA hypermethylation of *HOTAIR* was previously shown to be connected to its expression^29^,^30^, further underlining the fact that from DNA methylation data alone transcriptional activation of a gene cannot be unequivocally deduced.

**Figure 5:**
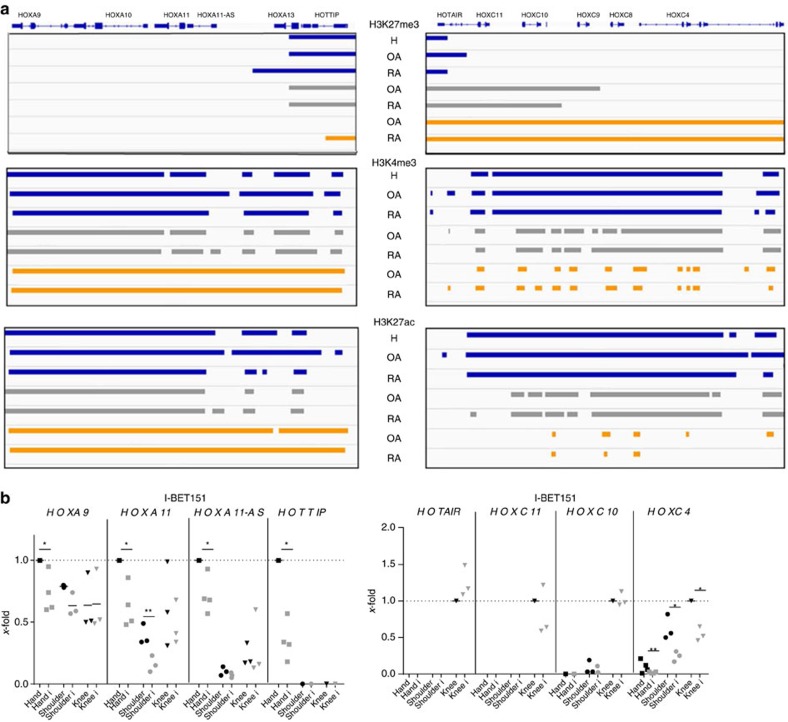
Joint-specific histone marks in HOXA and HOXC clusters in SFs. (**a**) Regulation of the positional HOX signatures in knee (blue), shoulder (grey) and hand (orange) SFs by histone modifications. H3K27me3, H3K4me3 and H3K27ac marks were measured by chromatin immunoprecipitation DNA sequencing (ChIP-seq) and significant enrichment calculated by the peak calling algorithm SICER. Data are visualized using Integrative Genomics Viewer (IGV) (**b**). Joint-specific effects of I-BET151, an inhibitor of BET reader proteins, on the expression of HOXA and C transcripts in hand (*n*=4), shoulder (*n*=3) and knee (*n*=3) RA SFs (*x*-fold calculations; expression in hand and knee RA SFs, respectively, was set to 1). Statistics: one-sample *t-*test or two-tailed paired *t-*test, *P<0.05; **P<0.01.

Temporal^28^ and spatial^31^ collinear activation of HOX genes during patterning of rostrocaudal axis in mouse embryos is closely associated with the successive removal of repressive H3 lysine 27 trimethylation (H3K27me3) marks and increased deposition of activating H3K4me3 and H3 acetylation (H3ac) marks in actively transcribed HOX chromatin domains. Analysis of these histone marks by chromatin immunoprecipitation (ChIP) DNA-sequencing followed by visualization of significantly enriched numbers of mapped reads using SICER^32^ showed an increase of repressive H3K27me3 marks in knee and shoulder compared with hand SFs in the *HOXA13* locus. This was accompanied by less enriched active H3K4me3 and H3K27ac marks in knee and shoulder across the 5′ end of HOXA (Fig. 5a; coverage plots are shown in Supplementary Fig. 4). In HOXC, H3K27me3 marks were mostly absent and the active marks enriched in knee SFs corresponding to the high expression of 5′ HOXC-encoded transcripts in these joints. In shoulder SFs the absolute H3K27me3 enrichment was decreasing and active marks increasing from the 5′ to 3′ end of the HOXC locus, coinciding with gradual increase of 3′ HOXC transcription in these joints. In hand SFs the whole locus was enriched for H3K27me3 and active marks were only enriched at single peaks (Fig. 5a).

To assess the reader proteins of joint-specific HOX marks, we treated SFs from different joints with I-BET151, a selective inhibitor of the bromodomain and extra-terminal (BET) protein family members BRD2, BRD3 and BRD4. Proteins of the BET bromodomain family bind ɛ-*N*-acetylated lysines of histone 3 (H3) and H4, thereby acting as reader proteins for histone acetylation at actively transcribed sites^33^. BET inhibition significantly reduced the expression of transcripts encoded in the 5′ end of the HOXA cluster in hands and to a minor extent in shoulders, but not in knees (Fig. 5b). In contrast, the positionally regulated transcripts from the 5′ tip of the HOXC locus (*HOTAIR*, *HOXC11*), which are expressed exclusively in SFs from the lower extremities, were not affected (Fig. 5b). Only the expression of *HOXC4*, which lies at the 3′ end of the cluster, was dependent on BET reading.

These results show a complex and joint-specific relationship between DNA methylation, histone marks and reader proteins in regulating the transcriptional activation of the HOXA and HOXC locus in SFs at different anatomical locations.

### Arthritis-relevant pathways are positionally enriched

To explore whether positional transcriptional diversity of SFs translates into joint-specific and/or arthritis-relevant functional phenotypes of SFs, we conducted pathway enrichment analysis of RNA sequencing data by MetaCore using a two-step approach. First, we compared the joints of the upper versus lower extremities (hands and shoulders versus knees) to address the differences along the anteroposterior body axis (*nota bene*: spondyloarthropathies for instance affect predominantly lower extremity joints). In the second step, we conducted pairwise comparisons between different joints (hand versus knee, hand versus shoulder and shoulder versus knee). By doing this we assessed not only the specific contribution of hands and shoulders to the anteroposterior differences, but also explored pathway differences along the proximodistal limb axis. The list of positionally enriched process networks is available in Supplementary Data 3. Out of 16 process networks 12, which were enriched in the upper versus lower extremity comparison, appeared also enriched in the pairwise comparisons between hands and knees and/or shoulders and knees (anteroposterior axis). Of these, eight were common to hand versus knee and shoulder versus knee comparisons. Among networks uniquely enriched between joints, 2 were present in hand versus knee, 7 in shoulder versus knee and 10 in hand versus shoulder comparisons. This suggested a deep functional specialization of SFs along proximodistal limb axis (shoulder versus hand) and anteroposterior body axis (shoulder and hand versus knee).

**Figure 6:**
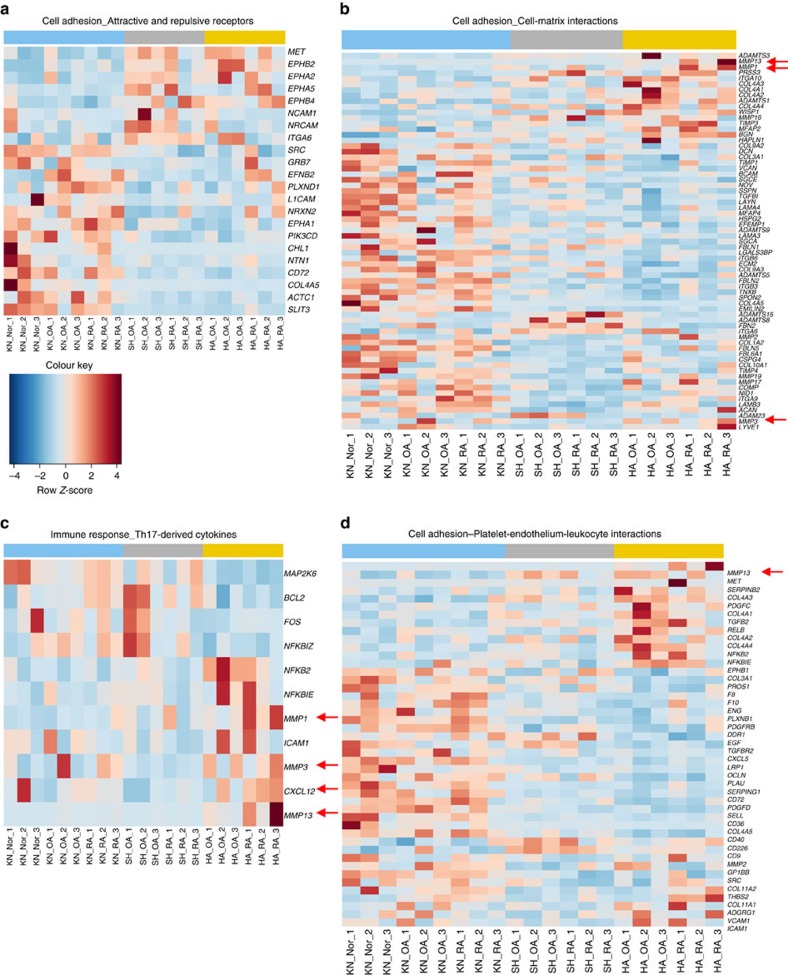
MetaCore pathway analysis of transcriptomes from different joints. Process Network Enrichment analysis of RNA sequencing data (*n*=21) between joint locations (knee versus hand, knee versus shoulder and hand versus shoulder) and upper versus lower extremities using MetaCore from Thomson Reuters (see also Supplementary Data 3). (**a**–**d**) Heatmaps of the enriched transcripts for selected arthritis-relevant process networks among all significantly enriched process networks between joints are shown. Red arrows point to the transcripts further studied or discussed in the paper. Heatmaps display *Z*-scores of normalized read counts.

Joint-specific networks, for example, ossification and bone remodelling (Supplementary Fig. 5a), were significantly enriched between different anatomical locations, pointing to anatomical molecular diversity at different joint locations. In addition, a number of arthritis-relevant networks (Fig. 6 and Supplementary Fig. 5b) were identified as positionally enriched between upper versus lower extremities and different joint locations, in particular networks regulating cell adhesion (Fig. 6a), cell–matrix interactions (Fig. 6b and Supplementary Fig. 5b), immune response (Fig. 6c), chemoattraction and cell–cell interactions (Fig. 6c,d and Supplementary Data 3). Chronic arthritis often takes a more destructive course when hand joints are affected, for example, the more aggressive subtype of erosive OA is almost exclusively found in interphalangeal joints of the hands^34^,^35^. In our enrichment analysis Proteolysis_Connective tissue degradation (Supplementary Fig. 5b) and cell adhesion_cell matrix interaction (Fig. 6b) networks showed clear enrichment in SFs from upper extremity joints versus knees and hands versus shoulders. *MMP1* (Fig. 6b and Supplementary Fig. 5b) and *MMP13* (Fig. 6b and Supplementary Fig. 5b), both of which contribute to cartilage degradation in chronic arthritis^20^, might confer stronger matrix degradation properties to upper versus lower extremity SFs and to distal versus proximal SFs, respectively.

### *HOTAIR* can modulate MMP1 expression

**Figure 7:**
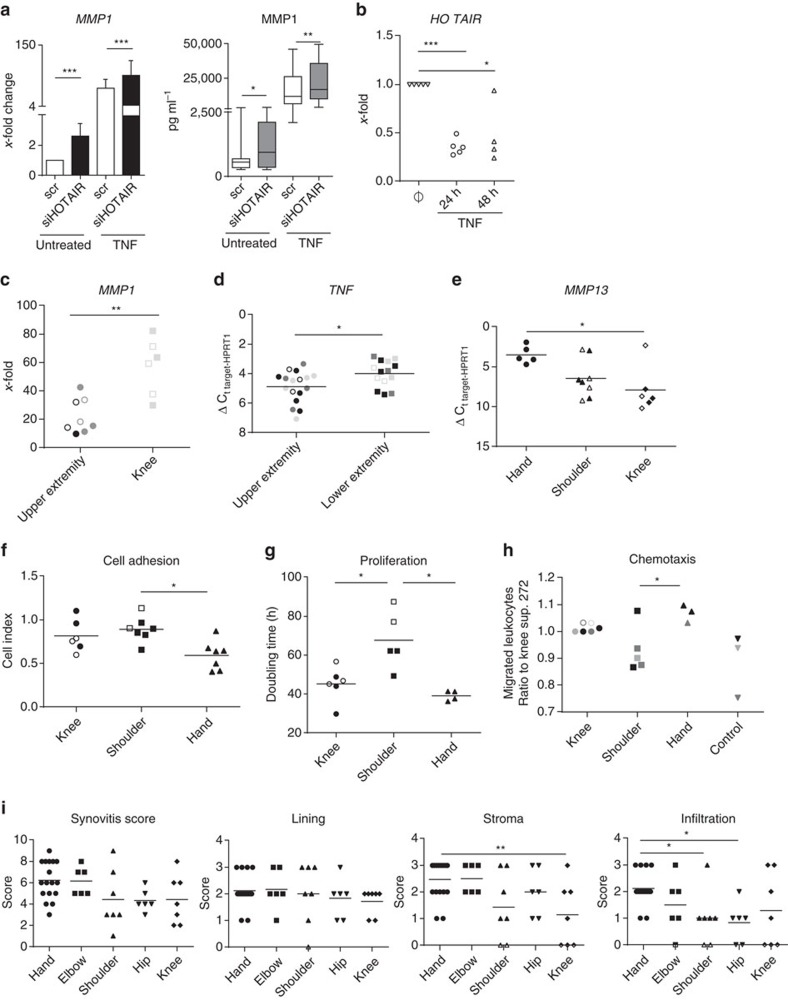
Functional analysis of SFs from different locations. (**a**) Increased constitutive and TNF-induced expression of *matrix metalloproteinase 1* (*MMP1*) mRNA (*n*=11, qPCR, mean±s.d., mean ΔCT±s.d.=9.79±0.94 of MMP1 in unsilenced, untreated knee SFs) and MMP1 protein (*n*=8, ELISA, box and whiskers: min to max) in knee OA SFs transfected with *HOTAIR* siRNA versus control siRNA (scr) for 72 h (TNF (10 ng ml^−1^) for 24 h). (**b**) Suppression of *HOTAIR* in knee SFs (*n*=5) by TNF (10 ng ml^−1^, 24 h) qPCR. (**c**) Increased *MMP1* production in response to TNF (10 ng ml^−1^) in knee SFs. Fold change *(x*-fold) of *MMP1* in TNF-stimulated (24 h) versus unstimulated SFs from upper extremity joints (hands: dark grey; elbows: light grey; shoulders: black) and knees, qPCR. (**d**) Higher expression of *TNF* in synovial tissues from lower extremity (ankles: dark grey; knees: light grey; hips: black) than from upper extremity joints (hands: dark grey; elbows: light grey; shoulders: black), qPCR. (**e**) Increased *MMP13* expression in hand compared with knee synovial tissues, qPCR. (**f**) Increased adhesion to culture wells (xCELLigence system) of shoulder versus hand SFs. (**g**) Lower proliferative potential of shoulder versus hand and knee SFs (doubling time (hours), xCELLigence system). (**h**) Increased leukocyte migration towards supernatants of hand versus shoulder SFs. Number of leukocytes migrating towards supernatants from SFs of different locations or towards medium. Data were normalized to the number of SFs migrating towards the supernatant from one knee SF. Healthy donors are denoted with black or grey shapes. (**i**) Increased leukocyte infiltration in the synovium from hands versus shoulders and hips. Increased density of synovial stroma in hands versus knees. Haematoxylin/eosin-stained RA synovial tissues from different locations (see Supplementary Table 5) were scored with a synovitis score^38^, composed of changes in stroma, synovial lining thickness and leukocyte infiltration. Statistics: paired (**a** (qPCR), **b**) or unpaired (**c**,**d**) *t*-test, two-tailed Wilcoxon matched-pairs signed rank test (**a** (ELISA)), one-way analysis of variance (ANOVA) with Bonferroni's (**e**–**h**) or Dunn's (**i**) post tests. *P<0.05; **P<0.01, ***P<0.001. Open shapes are OA SFs and closed shapes are RA SFs.

The lncRNA *HOTAIR* regulates the expression of hundreds of genomic loci by recruitment of polycomb repressive complex 2 (PRC2) and lysine-specific demethylase 1 (LSD1) for concomitant H3K27 methylation/H3K4 demethylation and transcriptional repression^36^. Due to the widespread effects of *HOTAIR* on gene transcription^37^ and its sharp lower versus upper extremity expression pattern in SFs, we explored the potential contribution of positional *HOTAIR* expression to the positional regulation of *MMP1* (higher in upper versus lower extremity, Fig. 6b,c and Supplementary Fig. 5b) in SFs. Silencing of *HOTAIR* in knee SFs, which have endogenously high *HOTAIR* expression, significantly increased basal and TNF-induced MMP1 mRNA and protein expression (Fig. 7a), showing that *HOTAIR* can restrict MMP1 production in knee SFs (Fig. 6a using short interfering RNA (siRNA), Supplementary Fig. 6 using GapmeR). Stimulation of knee SFs with TNF significantly repressed *HOTAIR* expression (Fig. 7b), indicating that inflammation-driven decrease of positional *HOTAIR* expression can facilitate matrix-destructive behaviour of lower extremity SFs.

These experiments show that a positionally expressed lncRNA in the HOX cluster can be influenced by an inflammatory environment that might contribute to activate joint destructive and inflammatory pathways in a location-specific manner.

### Positional transcriptomes translate into specific phenotypes

TNF is a major pro-inflammatory cytokine in the pathogenesis of RA and therapeutic anti-TNF antibodies have become an important pillar for successful RA management. The induction of MMP1 in response to TNF was significantly larger in knee compared with upper extremity SFs (Fig. 7c), showing that not only basal expression of arthritis-relevant molecules, but also the response to TNF differs between joints. TNF-induced decrease of *HOTAIR* could also play a role in the stronger induction of MMP1 in response to TNF in knee compared with upper extremity SFs (Fig. 7b). *In vivo*, TNF levels were higher in synovial tissues from lower extremity joints compared with synovial tissues from upper extremity joints (Fig. 7d). Furthermore, measurements of MMP expression in whole synovial tissue explants showed that RA synovial tissues from hand joints expressed significantly more *MMP13* than synovial tissues from knees of RA or OA patients (Fig. 7e). However, there was no difference in *MMP1* or *MMP3* production between hand, shoulder and knee synovial tissues (Supplementary Fig. 7). The higher basal expression of *MMP1* in hand SFs might therefore be levelled out by the increased amounts of TNF and the stronger response to TNF in knee joints.

In cultured cells, real-time analysis of proliferation and adhesion of SFs to tissue culture wells revealed that shoulder SFs exhibit more pronounced adhesive characteristics compared with hand SFs (Fig. 7f), while knee and hand SFs have significantly higher proliferative potential (Fig. 7g). Functional studies in SFs confirmed the enhanced chemotactic characteristics of hand and knee compared with shoulder SFs (Fig. 7h). Increased production of *CXCL12* by hand SFs might contribute to the increased chemotactic properties (Fig. 6c). Furthermore, scoring synovial tissue sections from different joints of RA patients (Supplementary Table 5) with an established synovitis score (Krenn score)^38^ showed that the infiltration of immune cells is significantly higher in synovium from hands versus shoulders or hips (Fig. 7i). The density of the synovial stroma was significantly different between the analysed RA joints, in particular hands and knees (Fig. 7i).

All in all, this analysis suggests that RA might manifest differently at different joint locations that might be exploited in the design of joint-specific therapeutic approaches.

## Discussion

While fibroblasts from different tissues were long considered as functionally homogenous cells, involved primarily in extracellular matrix production, it is now accepted that these cells perform a number of specialized functions^9^,^39^,^40^. The crucial role of cancer-associated fibroblasts in cancerogenesis^41^ and of SFs in arthritis^11^ is widely acknowledged.

Our work demonstrates that SFs from different anatomical locations exhibit significant differences in transcriptome, epigenome and function. Previous studies have recognized differences between hip and knee joints, mainly in the context of OA. In particular, DNA methylation patterns in chondrocytes and SFs were found to differ between these two joint locations^42^,^43^,^44^. Here we study SFs from a large number of joint locations, including small joints of the hands and feet, that are characteristically and severely affected by RA. We show that transcriptional and epigenetic differences are not confined to hip and knee SFs exclusively, but include all joint locations along limb axes, and follow a pattern that was imprinted during embryonic development. Although we could obtain non-arthritic human SFs only from knee joints, the conservation of principal positional genes between species and diseases shows that a number of positional gene signatures in SFs are arthritis independent. Still, it can be expected that some of the site-specific differences in gene expression reflect disease-specific mechanisms in SFs that differ in different joints, as suggested recently^43^. We demonstrate that the transcriptional diversity of SFs translates into pronounced functional diversity, thereby creating a unique microenvironment for each joint. Future studies should endeavour to answer in how far these distinct microenvironments at different anatomical location influence arthritis development.

As in dermal fibroblasts^5^, positional HOX gene signatures are sufficient to discriminate SFs from different anatomical locations. We demonstrate that DNA methylation and histone marks tightly regulate joint-specific HOX gene expression in SFs. The role of epigenetics in positional HOX gene regulation has previously been established during embryogenesis^1^,^28^,^45^ and in specific adult cells and tissues^22^,^42^,^46^,^47^. To our knowledge, this is the first study to demonstrate that BET reading is involved in the transcriptional regulation of specific HOX genes, in particular in a joint-specific manner. In accordance with this, alleles of *fs(1)h*, a homologue BET protein in *Drosophila melanogaster*, were shown to cause segmental abnormalities^48^. BET proteins, unlike other acetylated histone-binding proteins, remain associated with acetylated histones during mitosis and are likely to contribute to the transmission of transcriptional memory during cell division^49^,^50^. Our results show that inhibition of BET reading by I-BET151 in hand SFs reduces the expression of all measured 5′ HOXA transcripts with the strongest repression of *HOTTIP*. *HOTTIP* is a key locus control element of 5′ HOXA genes and distal identity, coordinating 5′ HOX gene activation by maintaining H3K4me3 across the 5′ end of the cluster^21^. Hence, in addition to directly inhibiting BET reading, I-BET151 might repress the 5′ HOXA transcription in distal SFs via reduction of *HOTTIP* expression. Furthermore, *HOTTIP* might integrate the roles of histone acetylation and histone methylation to regulate the transcription of the 5′ HOXA-encoded genes.

Despite the widely studied role of HOX genes in tissue regeneration, repair^51^ and human malignancies^52^, the understanding of whether positional stromal transcriptional programs confer site-specific susceptibility of adult tissues to a particular disease is only beginning to be elucidated^7^,^8^,^47^. A recent study demonstrated that distinct positional HOX signatures pre-imprint aortic vascular smooth muscle cells to differentially respond to TNF, partly explaining the characteristic segmental susceptibility of aorta to atherosclerosis^7^. Likewise, a distinct positional signature of developmental genes, including HOX genes, was associated with the limited inflammatory nature of lower body adipose tissues, explicating reduced metabolic complications in subjects with lower body fat accumulation^8^. Apart from the HOX genes, a recent study reported that the outcome of *KRAS* mutations and the status of DNA mismatch repair genes in patients with stage III colon cancer should be interpreted in the context of the primary tumour location with regard to the proximal versus distal colon^53^. Our results demonstrate that SFs from different joints are functionally specialized cells not only differentially responding to pro-inflammatory stimuli (TNF), but also exhibiting distinct adhesive, proliferative, chemotactic and matrix destructive characteristics. In particular, hand SFs display prominent chemotactic and matrix-destructive characteristics that may explain why inflammatory arthritis tends to be more aggressive and destructive in hands compared with other joints.

In conclusion, our findings deeply alter the current understanding of synovial biology in health and disease. The existence of positionally imprinted ‘risk' signatures of SFs provides a mechanistic insight into the early stages of joint pathologies and might prove critical for understanding late-stage arthritis—its chronicity, spreading and lack of therapeutic response. Our data offer a concept that the local identity of stromal cells predisposes to development of positional disease patterns, not only in arthritis but in any disease with a prominent stromal component. Anatomical location undoubtedly matters in disease development and should be considered in future in diagnosis, treatment and prognosis.

## Methods

### Patients and cell culture

Synovial tissues from joints of hands, elbows, shoulders, hips and knees were obtained from OA and RA patients undergoing joint replacement surgery at the Schulthess Clinic Zurich, Switzerland. Synovial biopsies from knees of patients experiencing joint pain without inflammation or cartilage destruction (healthy) and from ankles of patients with resolving arthritis were gathered at the Queen Elizabeth Hospital in Birmingham, UK. RA patients fulfilled the 2010 ACR/EULAR (American College of Rheumatology/European League Against Rheumatism) criteria for the classification of RA^54^. The studies were approved by the local ethic committees of the University Hospital Zurich, Switzerland, and the University of Birmingham, UK. Informed consent was obtained from all patients. Snap frozen synovial tissues were minced and RNA was isolated using TRIzol (Life Technologies) according to the manufacturer's protocol followed by DNA digestion and purification with miRNeasy Mini kit (Qiagen). Synovial tissues were digested with dispase (37 °C, 1 h) and SFs were cultured in Dulbecco's modified Eagle's medium (DMEM; Life Technologies) supplemented with 10% fetal calf serum (FCS), 50 U ml^−1^ penicillin/streptomycin, 2 mM L-glutamine, 10 mM HEPES and 0.2% amphotericin B (all from Life Technologies). Purity of synovial fibroblast cultures was confirmed by flow cytometry showing the presence of the fibroblast surface marker CD90 (Thy-1) and the absence of leukocytes (CD45), macrophages (CD14; CD68), T lymphocytes (CD3), B lymphocytes (CD19) and endothelial cells (CD31). Cell cultures were negative for mycoplasma contamination as assessed by MycoAlert mycoplasma detection kit (Lonza). SFs from passages 5 to 8 were used for deep RNA sequencing and functional studies. Total RNA from SFs was isolated with the miRNeasy Mini kit (Qiagen) including on-column DNAaseI digestion.

### Animals and cell culture

Wild-type and TNF transgenic (TghuTNF) C57BL/6 mice (*n*=10 each, see Supplementary Table 4) were generated as previously described^26^ and housed under specific pathogen-free conditions in the animal facilities of the Biomedical Sciences Research Centre (BSRC) Alexander Fleming. All experiments were approved by the local ethics committee and in accordance with the institutional care and use committee of BSRC Alexander Fleming. Fully diseased TghuTNF and healthy wild-type mice were killed at 6–8 weeks of age. The sample size for animal experiments was chosen based on previous laboratory experience and published evidence from other groups. A sample size larger than five animals is usually adequate for analysing responses of mouse SFs. No randomization was used and no blinding was done. TghuTNF animals have a 100% penetrance of joint disease. At the age of 6–8 weeks, joint disease is considered as established. Mice were randomly selected from three individual breedings (TghuTNF and wild-type controls) and each group was co-housed. TghuTNF and wild-type littermate controls were randomly chosen from the litters. Mouse fibroblasts were isolated from ankles, knees and front paws (see Supplementary Table 4) as described in ref. 55. The purity of mouse SFs was determined as follows: >80% CD90.2+ and <5% CD11b+, with >90% Vcam1+ within CD90.2+ population. The cultures of SFs with lower purity were excluded from further analysis (see Supplementary Table 4). Total RNA from mouse SFs was isolated using TRIzol as described above.

### Treatment of SFs

SFs from RA patients were treated with the BET bromodomain inhibitor I-BET151 hydrochloride (1 μM; Tocris Bioscience) or equal amounts of dimethyl sulfoxide (control treatment) for 24 h as previously described^56^. I-BET151 treatment did not affect cell viability or impaired receptor-proximal signalling events^56^. RNA was isolated from SFs using the ReliaPrep RNA Cell Miniprep System (Promega), including on-column DNaseI digestion.

### RNA sequencing of healthy and arthritic SFs

Total RNA from SF (RNA integrity number ≥9.5, *n*=21, patient's characteristics in Supplementary Table 1) was used for generation of both long and small RNA libraries for sequencing. The RNA quantity and quality were evaluated using the Qubit RNA BR Assay Kit (Life Technologies) and the Agilent RNA 6000 Nano kit with Agilent 2100 Bioanalyzer instrument (Agilent Technologies, Inc.), respectively.

The Illumina TruSeq Stranded total RNA protocol with the TruSeq Stranded total RNA Sample Preparation Kit was used to produce the long RNA-seq libraries from 550 ng of total RNA as follows: (1) ribosomal RNA removal with human-specific probes (Epicentre); (2) fragmentation of enriched RNA; (3) first-strand complementary DNA (cDNA) synthesis using reverse transcriptase and random hexamers; (4) second-strand cDNA synthesis with DNA polymerase; (5) adenylation of 3′ ends of the blunt fragments; (6) ligation of multiple indexing adapter to the ends of cDNA; and (7) PCR amplification of cDNA to enrich DNA fragments having adapter molecules on both ends and to amplify the amount of DNA in the library. The Illumina TruSeq Small RNA protocol was used to produce the small RNA-seq library from 1 μg of total RNA as follows: (1) ligation of the RNA 3′ and RNA 5′ adapters to the sample; (2) reverse transcription to create single-stranded cDNA; (3) PCR amplification of cDNA with a common primer and a primer containing one of 48 index sequences; and (4) gel excision (between 145 and 160 bp) and gel purification of amplified cDNA construct. The quality and quantity of the generated libraries were determined by Agilent Technologies 2100 Bioanalyzer with DNA-specific chip and quantitative PCR (qPCR) using Illumina adapter-specific primers using the Roche LightCycler system (Roche Diagnostics), respectively.

Diluted indexed long RNA-seq (10 nM) and small RNA-seq (1 nM) libraries were pooled in equal volumes, used for cluster generation (TruSeq SR Cluster Kit v3-cBot-HS reagents, according to the manufacturer's recommendations) and sequenced (TruSeq SBS Kit v3-HS reagents, Illumina HiSeq 2500, the high output mode with single read approaches: 100 bp (long RNA-seq) and 50 bp (small RNA-seq)).

### Bioinformatics analysis of RNA sequencing data

For protein coding RNA and lncRNA, sequencing data reads were quality-checked with FastQC (http://www.bioinformatics.babraham.ac.uk/projects/fastqc/). Reads were trimmed with Trimmomatic (4 bases hard-trimming from the start, and adapter trimming at the end). We aligned the trimmed reads to the reference genome and transcriptome (FASTA and GTF files, respectively, Ensembl GRCh37) with STAR version 2.5.1b (ref. 57). The average number of high-quality reads, reads aligned and reads uniquely aligned per sample were 33.8, 33.2 and 28.6 million, respectively, for ribo-depleted libraries. Gene expression was quantified using the R/Bioconductor package Rsubread^58^ version 1.22. Differentially expressed genes between joints and diagnoses were identified using the R/Bioconductor packages DESeq2 (ref. 59) version 1.12.4. For each comparison the set of differentially expressed genes was identified as genes with an absolute value of the log2 ratio >0.5 and a *P* value <0.05. Heatmaps and sample clustering are based on DESeq2 normalized values. Heatmaps display *Z*-scores of normalized read counts.

For the small RNA experiment, ncPRO-seq with default settings was used to quality control (QC), map and quantify the abundances of microRNA^60^. The average number of trimmed reads for small RNA libraries was 11.5 million reads. Differentially expressed microRNA between joints and diagnoses were identified using the R/Bioconductor packages edgeR^61^ version 3.14.0. For each comparison the set of differentially expressed microRNA was identified as the microRNAs with an absolute value of the log2 ratio >0.5 and a *P* value <0.05. Heatmaps and clustering of microRNAs are based on the expression counts normalized with edgeR TMM method. Heatmaps display *Z*-scores of normalized read counts.

The unsupervised hierarchical cluster analysis of RNA-seq data (Fig. 1) is based on normalized reads after log transformation with an offset of 10 counts added. The 100 genes (protein coding, long noncoding and microRNA) with the biggest variance across all samples were used.

Supervised clustering was based on the union of genes found differentially expressed in each individual comparison. From each comparison the 25 most significant genes (satisfying a minimum log2 difference of 0.5 and maximum *P* value of 0.05) were used. Heatmaps highlighting the discriminating power of HOX genes (Fig. 2a–c) are based on the clustering of the transcripts encoded in HOXA, HOXB, HOXC and HOXD clusters (microRNAs were normalized by edgeR and protein coding and noncoding genes were normalized by DESeq2). Genes encoded within HOX loci were identified using the HOX gene annotation in GRCh38.p2, Ensembl Release 80 (May 2015)^62^. Only transcripts expressed above the threshold level were used in the cluster analysis.

Pathway enrichment analyses of genes differentially expressed between joint localizations (knee versus hand, knee versus shoulder and hand versus shoulder) were performed with MetaCore from Thomson Reuters (version 6.22, build 67265) using MetaCore ontologies including Pathway Maps, Process Networks, Diseases (by Biomarkers) and GO Processes and default settings (threshold of log (fold change)=0.5, *P* value=0.05, upregulated and downregulated genes both included). We reported process networks that are significantly enriched for differentially expressed genes and show the *P* value (<0.05) and false discovery rate (FDR <0.05) as provided by MetaCore (Supplementary Data 3). The heatmaps in Fig. 6 and Supplementary Fig. 5 show the differentially expressed genes that are contained in the respective network (colours represent *Z*-scores of normalized read counts). Each of the selected arthritis-relevant and joint-specific Process Networks is present among the significantly enriched Network Processes between joints.

### Quantitative real-time PCR

Total RNA was transcribed using random hexamers for mRNA and lncRNA or target-specific stem-loop reverse transcription primers (Life Technologies) for microRNA followed by real-time PCR (7500 or 7900HT real-time PCR system, Life Technologies) using TaqMan assays for microRNA (Life Technologies) and self-designed primers (see Supplementary Tables 6 and 7) in combination with SYBR green or TaqMan probes for mRNA and lncRNA. No template control samples, dissociation curves and samples containing the untranscribed RNA were measured in parallel. Data were analysed with the comparative C_T_ methods and presented as ΔCT or 2^−ΔΔCT^ as described in ref. 63. For normalization of mRNA and lncRNA expression *HPRT1* was used in synovial tissues and I-BET151 experiments and *beta2-microglobulin* in human and mouse SFs. The expression of microRNA was normalized to *RNU6B* in human SFs and synovial tissues and to *snoU6* in mouse SFs. Heatmaps of HOX transcripts (Fig. 3) are based on the log2 ratios of sample ΔCT (CT target gene−CT endogenous control gene) expression values relative to the average ΔCT of a gene across all the samples as measured by qPCR. Supplementary Data 2 shows ΔCT values of HOX genes used to generate the relative expression heatmaps in Fig. 3.

### HumanMethylation 450 BeadChip

DNA was isolated from SFs (QIAamp DNA blood kit, Qiagen) obtained from hands (*n*=1 OA/4 RA), shoulders (*n*=3 OA/4 OA) and knees (*n*=1 OA/4 RA) of RA and OA patients. Samples were subjected to the Illumina HumanMethylation 450 array. After QC, we calculated differentially methylated CpG sites and islands using the COHCAP Bioconductor package (version 3.3) in R statistical program. First, we calculated the β-values for each CpG site from the array using the minfi package. Second, QC of the data was performed and principal component analysis plots from the sample β-values were generated with the COHCAP algorithm. Last, the CpG sites were defined as methylated if they had β-values of >0.7 and unmethylated if they had β-values of <0.3. In addition, we calculated the delta-β-value for each pair of joints and identified differential methylated sites as significant between joints with *P*<0.05 and FDR <0.05. Differentially methylated CpG sites were calculated using analysis of variance F-statistic for averaged β-values per group of samples (hand–knee, hand–shoulder and shoulder–knee). The FDR values were calculated using Benjamini and Hochberg test. For data visualization, we generated average wig files for each anatomical location and graphs using the tools from the University of California Santa Cruz (UCSC) Genome Browser.

### ChIP DNA sequencing

Chromatin was prepared on pellets of fixed cells from OA and RA knee, shoulder and hand SFs (*n*=1 per each diagnosis and location) and normal knee SFs (*n*=1) using the iDeal ChIP seq kit for Histones (Diagenode) with a shearing of 12 cycles (30′′ON 30′′OFF, Bioruptor Pico). The shearing efficiency was analysed using an automated capillary electrophoresis system Fragment Analyser (High sensitivity NGS fragment kit) after RNase treatment, reversion of crosslinking and purification of DNA. ChIP assays were performed using 1 million cells per IP and the following antibodies: H3K4me3 (0.5 μg, C15411003, Diagenode), H3K27me3 (1 μg, C15410195, Diagenode) and H3K27ac (1 μg, C15410196, Diagenode). A control library was processed in parallel using the same amount of control Diagenode ChIP'd DNA. Immunoprecipitated DNA was quantified by Qubit dsDNA HS kit and analysed by qPCR to evaluate the specificity of the IP (see Supplementary Table 8 for positive and negative control regions tested for each mark). Libraries were prepared from 1 ng of IP and input DNA using the MicroPLEX v2 protocol, quantified by BioAnalyzer, purified (AMPure beads) and eluted in TE. Purified libraries were quantified (Qubit ds DNA HS kit), analysed for size (Fragment Analyzer) and diluted to 20 nM concentration. Libraries were pooled followed by sequencing on an Illumina HiSeq 2500 (50 bp, single end) as follows: H3K4me3, 7 libraries per lane; input, 6 libraries per lane; H3K27ac, 5 libraries per lane; and H3K27me3, 4 libraries per lane. Quality of sequencing reads was assessed using FastQC, alignment was performed using bwa v. 0.7.5a and peak calling for different histone marks was performed using SICER v1.1 (ref. 32). The average±s.d. number of total reads per mark was as follows: H3K4me3 32.5±4.0 Mio, H3K27ac 41.4±4.4, H3K27me3 51.2±1.7 and input 43.4±15.6. Data were visualized using Integrative Genomics Viewer (IGV)^64^,^65^.

### *HOTAIR* silencing

Knee SFs were transfected with 25 nM siRNA targeting *HOTAIR* (Hs *HOTAIR* 3 siRNA, Cat. No. SI04446036, Qiagen, Sequence: 5′-CACGGAACCCATGGACTCATA-3′) or 50 nM antisense LNA *HOTAIR* GapmeR (Cat. No. 300600, Design ID 542251-1, Exiqon, Sequence: 5′-AGGCTTCTAAATCCGT-3′) using Lipofectamine 2000 (Invitrogen) according to the manufacturer's instructions. AllStars Negative siRNA control (25 nM, Qiagen) or Antisense LNA GapmeR Negative Control A (Cat No 300610) were used as transfection controls. At 24 h after transfection the medium was replaced and cells were lysed 48 and 72 h after GapmeR and siRNA transfection, respectively. In TNF stimulation experiments, 48 h after transfection with siRNA, SFs were stimulated with TNF (10 ng ml^−1^, R&D Systems, 210-TA-010) for additional 24 h, before supernatants were collected and RNA isolated for enzyme-linked immunosorbent assay (ELISA) and real-time PCR analysis. Transfection with si*HOTAIR* and *HOTAIR* GapmeR reduced the expression of *HOTAIR* in SFs for 48±21% (mean±s.d.) and 92% (median; min 62%—max 94%), respectively (Supplementary Fig. 6).

### Chemotaxis assay

Peripheral blood leukocytes were isolated using 8 volumes of hypotonic erythrocyte lysis buffer (ELB; 155 mM NH4Cl, 10 mM NaHCO3, 0.1 mM EDTA, pH 7.4) on fresh blood from healthy donors (diluted with 1 volume isotonic NaCl solution), incubation on ice for 5 min and centrifugation for 10 min at 350 × *g* at 4 °C. Cell pellets were resuspended in isotonic NaCl and again treated with ELB (4 volumes and then 1.5 volumes), washed twice with ice-cold phosphate-buffered saline, and resuspended in DMEM. Conditioned medium from SFs (100,000 cells, 1 ml of medium, 24 h) or control medium was added to the feeder tray of a 96-well transwell plate (5 mm polycarbonate membrane, Corning Costar). Leukocytes (1 × 10^6^ in 100 μl) were seeded air-bubble-free into the top inserts and allowed to migrate for 16 h. Then, 50 ml of ice-cold 20 mM EDTA/0.5% FCS in phosphate-buffered saline was added to the bottom wells and plates were incubated on ice for 15 min to facilitate detachment of any adhered cells. Migrated cells were counted with a CASY Cell Counter (Schärfe Systems). The ratio of migrated leukocytes for each donor was calculated as the number of cells migrating towards the conditioned or control medium normalized to the number of leukocytes migrating towards the conditioned medium from knee SFs, sample number 272. The experiment was performed three times using leukocytes from two healthy donors; leukocytes from the second healthy donor were isolated at two different time points.

### Synovitis score

Formalin-fixed, paraffin-embedded synovial tissues of RA patients (*n*=42, see Supplementary Table 5) were cut, put on slides and stained with haematoxylin/eosin. Synovitis was assessed by evaluation of the thickness of the lining cell layer, the cellular density of synovial stroma and leukocyte infiltration as described by Krenn *et al*.^38^.

### Real-time cell analysis (RTCA)

For RTCA of cell adhesion and proliferation of SFs, the xCELLigence RTCA DP Instrument (ACEA Biosciences, Inc.) was used. The 16-well E-plates were equilibrated with 100 μl of DMEM, 10% FCS for 30 min at room temperature. The impedance, expressed as arbitrary Cell Index (CI) units, of the wells with media alone (background impedance–Rb) was measured before adding the cells. SFs were detached with accutase, resuspended in DMEM 10% FCS, and seeded at cell density of 2,500 cells per well. Cell adhesion and spreading, measured as changes in impedance, was monitored every 5 min for a period of first 12 h and every 15 min after that for the next 12 h. The CI at each time point is defined as (Rn−Rb)/15, where Rn is the cell-electrode impedance of the well when it contains cells and Rb is the background impedance. Stable adhesion and spreading of SFs with no further fluctuations in CI were achieved at 16 h of measurement. At 24 h after seeding, 100 μl of medium per well in E-plates were exchanged with fresh 100 μl of medium. The proliferation of cells was monitored every 30 min for additional 90 h and was calculated as doubling time (CI=A × 2̂(*t*/CI doubling-time) in the period between 35 and 96 h after starting the measurement.

### Enzyme-linked immunosorbent assay

The concentration of MMP1 in supernatants of synovial fibroblasts transfected with *HOTAIR* siRNA or AllStars Negative siRNA control was measured using the human total MMP1 ELISA kit (R&D Systems, according to the manufacturer's instructions) and the GloMax-Multi+Detection System (Promega) with Instinct Software (Promega).

### Statistical analysis

Data were analysed with GraphPad Prism version 6.0 and IBM SPSS Statistics software. Data distribution was tested with Kolmogorov–Smirnov (small sample size) or D'Agostino and Pearson omnibus (large sample size) normality tests. The equality of variances was tested with Brown–Forsythe or Levene's tests. Multiple group comparisons were performed by analysis of variance (normal distribution) or Kruskal–Wallis (distribution not normal) test with adjustments for multiple comparisons using Bonferroni, Dunnett, Games-–owell (variances not equal) or Dunn's *post hoc* test. Paired samples were compared with two-tailed paired *t*-test or Wilcoxon matched-pairs signed rank test. One-sample *t*-test was used to compare *x*-fold change of treated versus control (set to theoretical mean of 1) groups. Unpaired *t*-test with Welch's correction was used when two samples with unequal variances were compared. Correlations were tested using Spearman's correlation coefficient. *P* value of <0.05 was considered statistically significant.

### Data availability

Sequence data that support this study have been deposited in European Nucleotide Archive (ENA) with the primary accession codes PRJEB14422 and PRJEB14595. Other data that support the findings of this study are available from the authors on request.

## Additional information

**How to cite this article:** Frank-Bertoncelj, M. *et al*. Epigenetically-driven anatomical diversity of synovial fibroblasts guides joint-specific fibroblast functions. *Nat. Commun.* **8,** 14852 doi: 10.1038/ncomms14852 (2017).

**Publisher's note:** Springer Nature remains neutral with regard to jurisdictional claims in published maps and institutional affiliations.

## Supplementary Material

Supplementary Information Supplementary Figures and Supplementary Tables

Supplementary Data 1 List of identified position and diagnosis specific transcripts by supervised cluster analysis of RNA sequencing data. Supervised cluster analysis of RNA sequencing data in synovial fibroblasts (n=21) of different anatomic locations and different diagnoses based on the union of genes found differentially expressed in each individual comparison. From each comparison the 25 most significant genes (satisfying a minimum log2 difference of 0.5 and maximum p-value of 0.05) were used.

Supplementary Data 2 Quantitative PCR of HOX transcripts in human and mouse synovial fibroblasts and synovial tissues. The values represent ΔCT values calculated as follows: ΔCT =CT of HOXtranscript - CT of endogenous control gene.

Supplementary Data 3 Joint specific and arthritis relevant process networks - enrichment analysis of RNA sequencing data. Provided as online Excel file. Process Networks Enrichment Analysis of RNA Sequencing Data (n=21) identifying significantly enriched process networks between upper and lower extremity joints and between specified joint locations (hand vs. shoulder, hand vs. knee, knee vs. shoulder) performed using MetaCore from Thomson Reuters and default settings (threshold of log (fold change) set to 0.5, pValue=0.05, FDR<0.05). FDR = False Discovery Rate.
