# Supplementary material for: Epigenetically-driven anatomical diversity of synovial fibroblasts guides joint-specific fibroblast functions
Source: Nat Commun. 2017 Mar 23;8:14852. doi: 10.1038/ncomms14852 (PMC5376654; doi:10.1038/ncomms14852)
Supplement: Supplementary Information — Supplementary Figures and Supplementary Tables [file ncomms14852-s1.pdf]

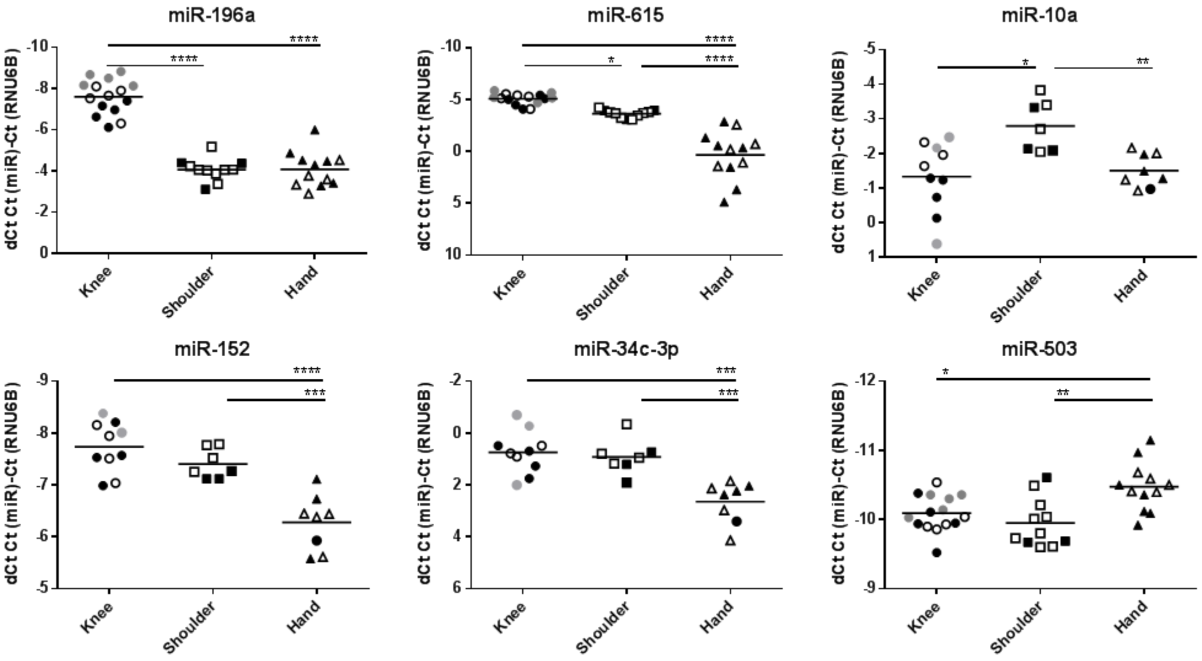

Supplementary Figure 1 MicroRNA expression in human synovial fibroblasts from different locations. MicroRNA, which were identified by RNAseq as most differentially expressed between human synovial fibroblasts (SF) from different joint locations were measured by qPCR in an independent set of rheumatoid arthritis (RA) and osteoarthritis (OA) SF isolated from knee, shoulder or hand joints. Open shapes are OA SF, closed shapes are RA SF. *=p<0.05; **=p<0.01; ***=p<0.001;****=p<0.0001 by two-way ANOVA with Bonferroni correction.

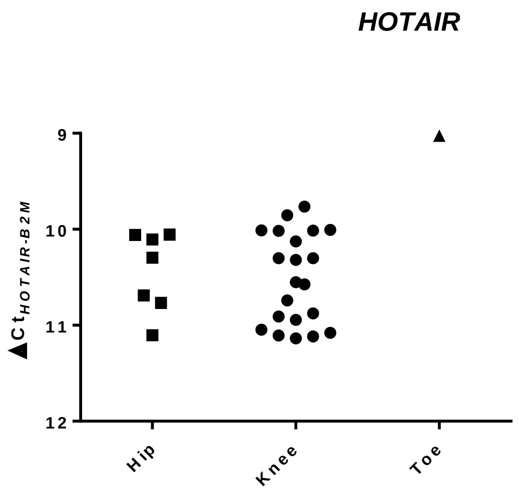

Supplementary Figure 2 The expression of *HOTAIR* in synovial fibroblasts from lower extremity joints. Human synovial fibroblasts (SF) from distal joint locations express more *HOTAIR* than SF from proximal joint locations (knee, hip). The expression of *HOTAIR* was measured by qPCR with normalization to *beta2 microglobulin (B2M)* in SF obtained from hip, knee and toe (proximal interphalangeal joints from digits II-IV) joints of patients with rheumatoid arthritis and osteoarthritis. Due to a limited access, a toe sample could be obtained from only one patient.

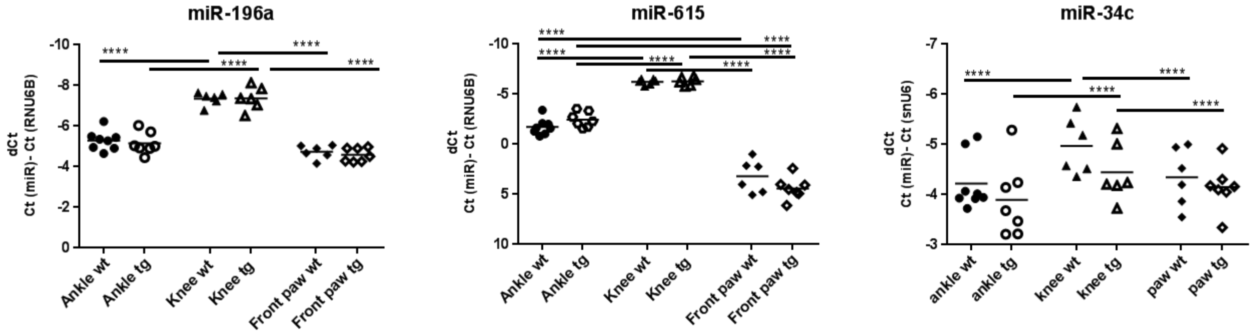

Supplementary Figure 3 MicroRNA expression in mouse synovial fibroblasts from different locations. MicroRNA, which were identified by RNAseq as most differentially expressed between human synovial fibroblasts (SF) from different joint locations were measured by qPCR in SF isolated from joints of wildtype (wt) and TNF transgenic (tg) C57BL/6 mice. ****=p<0.0001 by two-way ANOVA with Bonferroni correction.

H3K27me3 HOXA

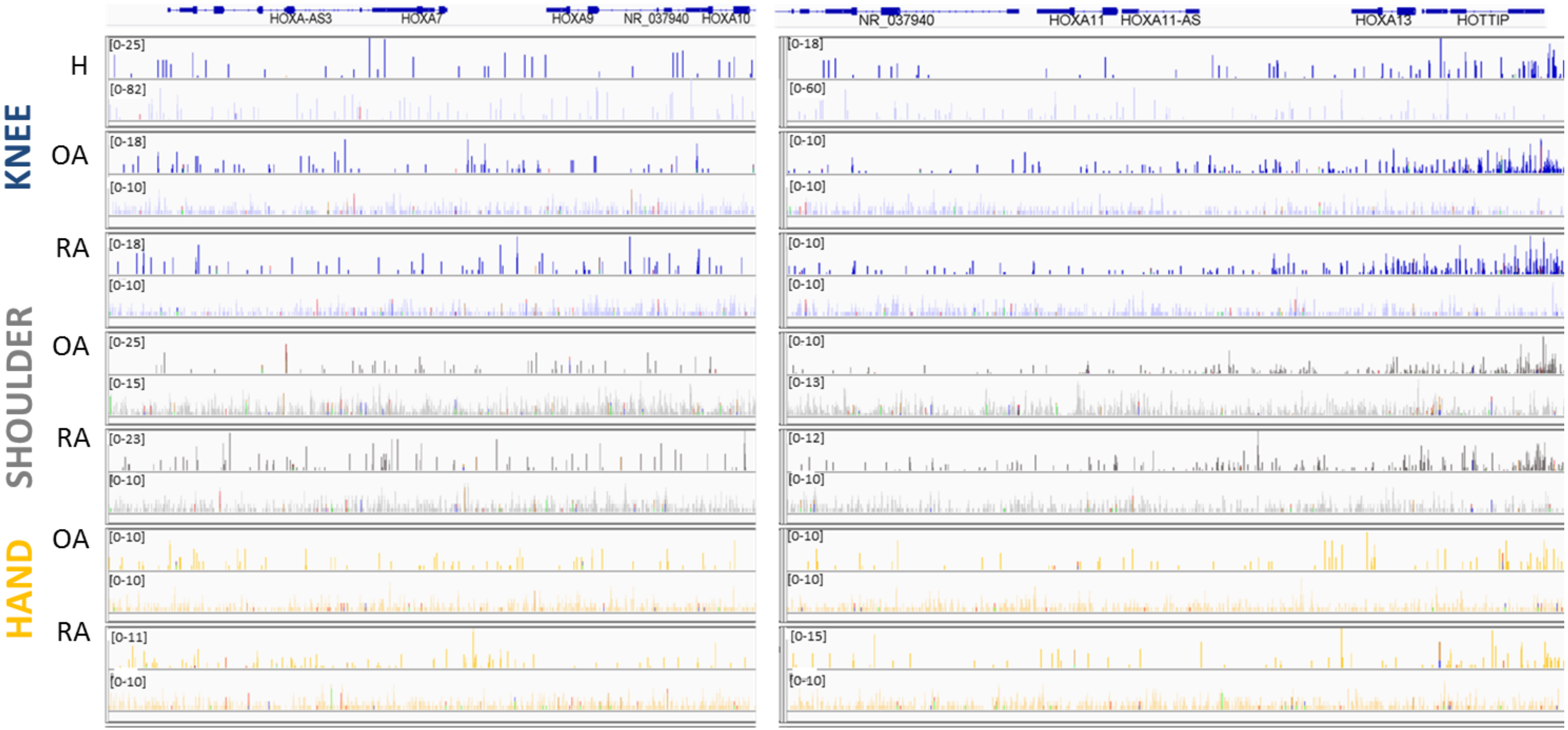

H3K27me3 HOXC

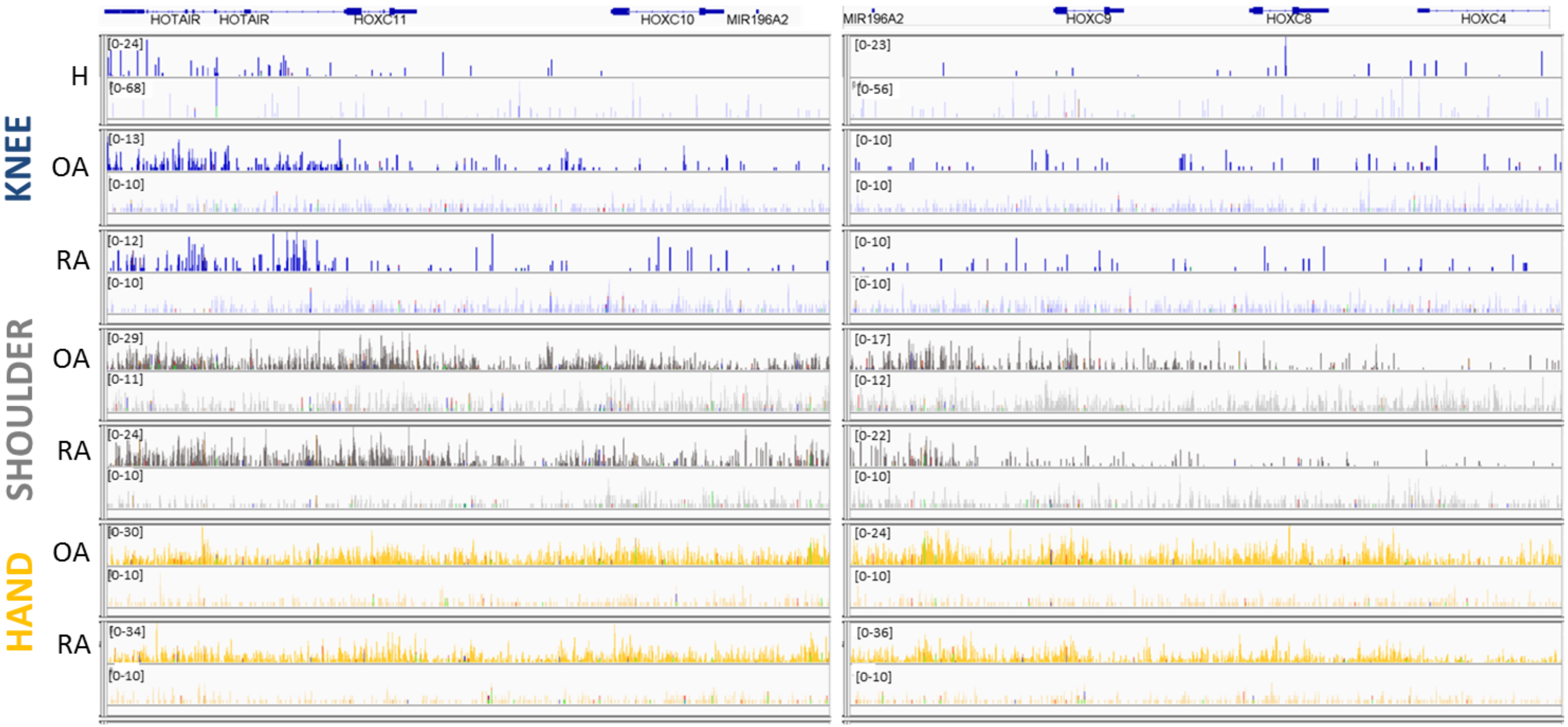

H3K4me3 HOXA

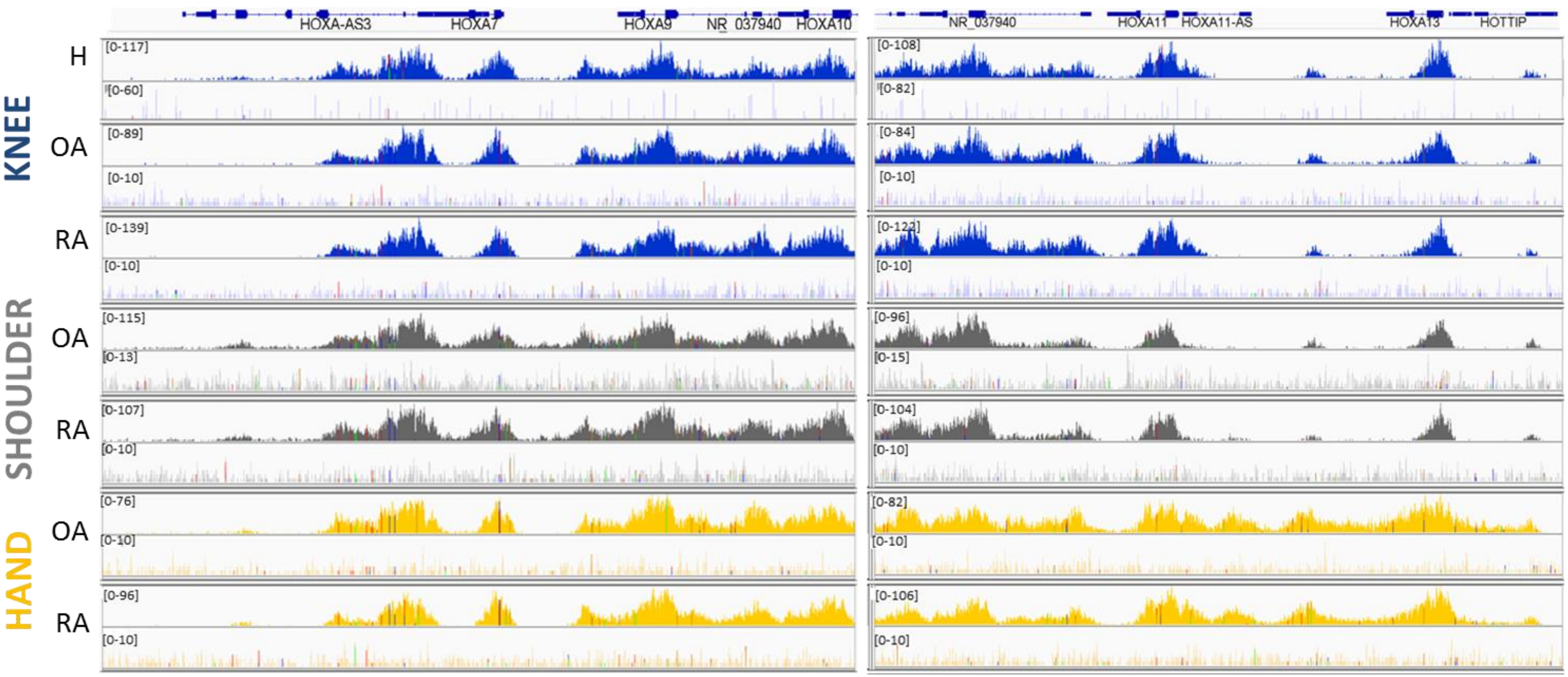

H3K4me3 HOXA

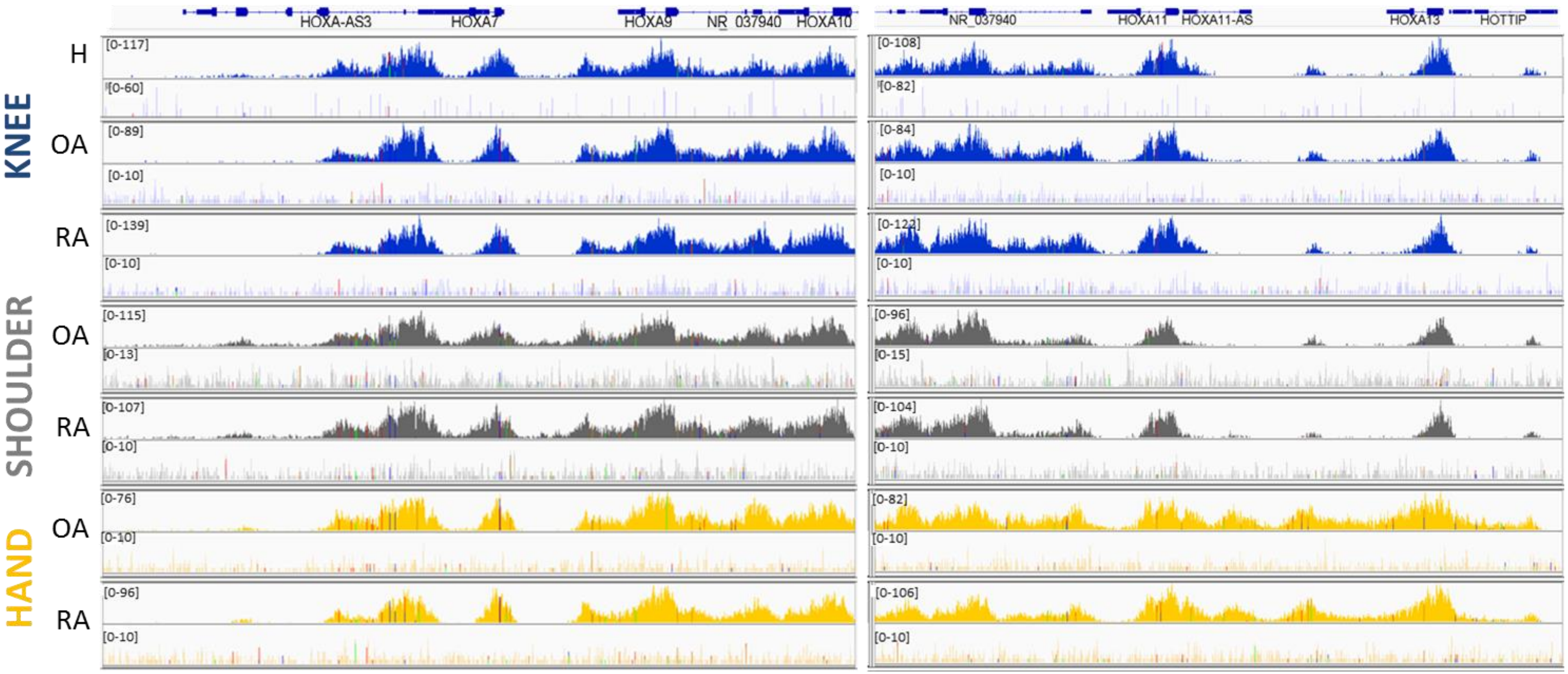

H3K4me3 HOXC

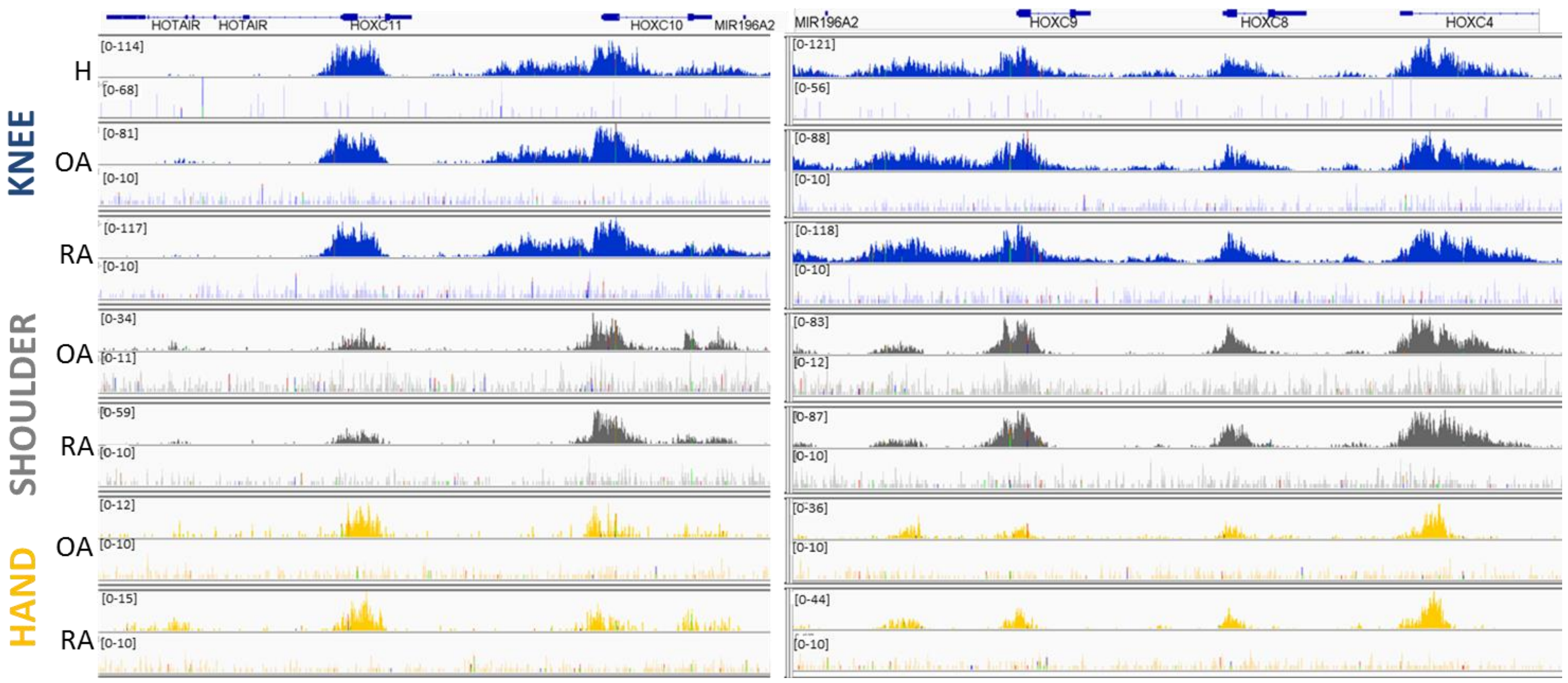

H3K27ac HOXA

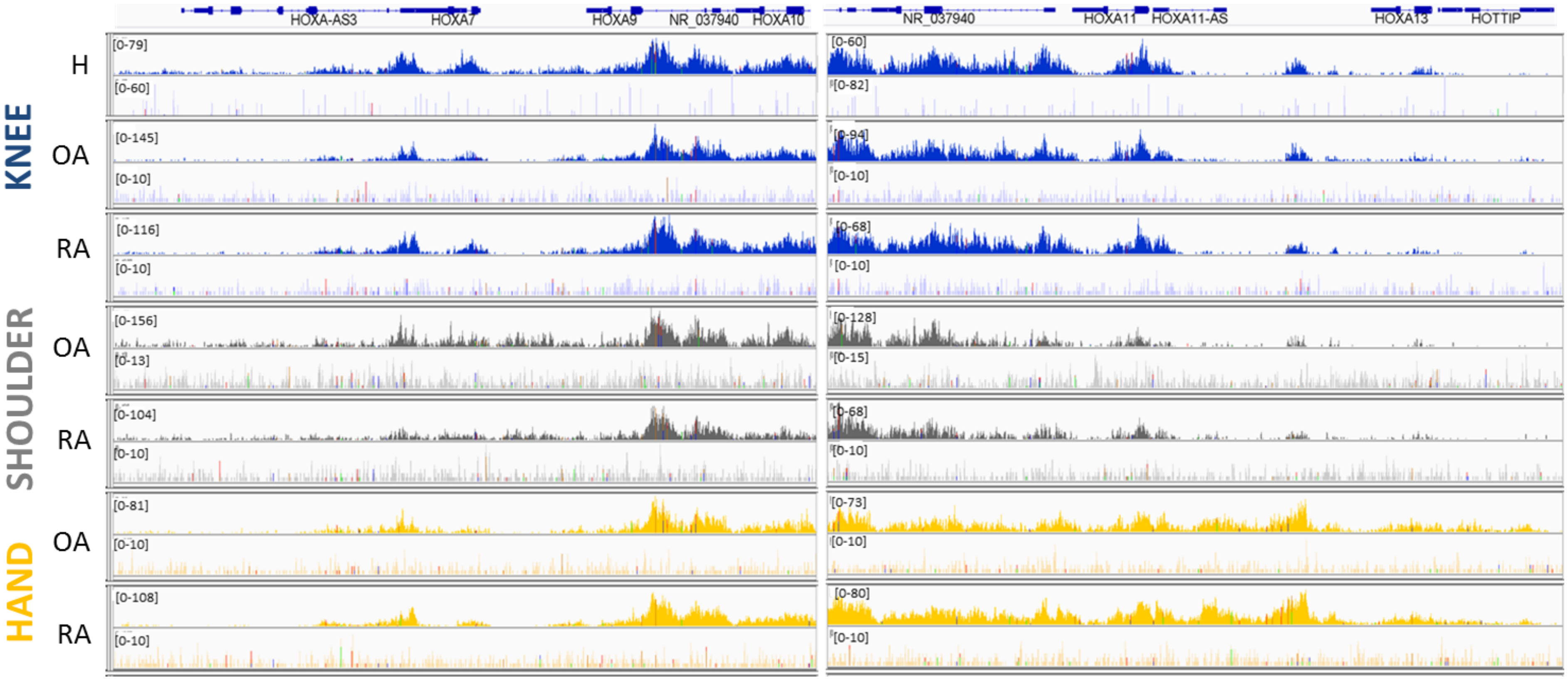

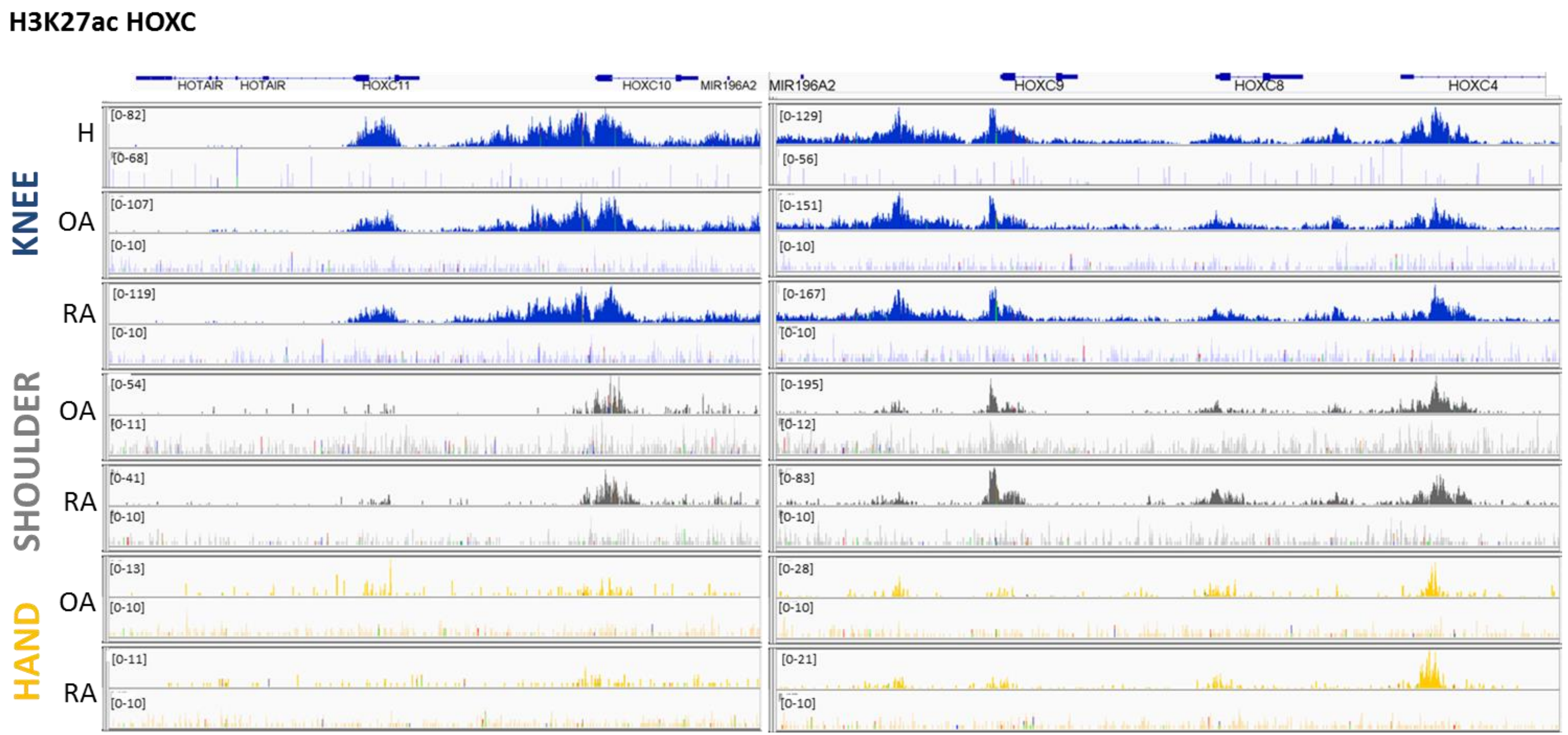

Supplementary Figure 4 Location specific histone marks in HOXA and HOXC clusters in synovial fibroblasts (SF). Regulation of the positional HOX signatures in knee (blue), shoulder (grey) and hand (orange) SF by histone modifications. H3K27me3, H3K4me3 and H3K27ac marks were measured by chromatin immunoprecipitation DNA sequencing (ChIPseq) in SF from knee, shoulder and hand joints of osteoarthritis (OA) and rheumatoid arthritis (RA) patients (n=1 each) and from knee of healthy (H) subject (n=1) with joint pain. Coverage values of H3K27me3, H3K4me3 and H3K27ac in HOXA and HOXC as measured by ChIP-Seq. Profiles of input samples are shown below each sample (light color). Data is visualized using Integrative Genomics Viewer (IGV).

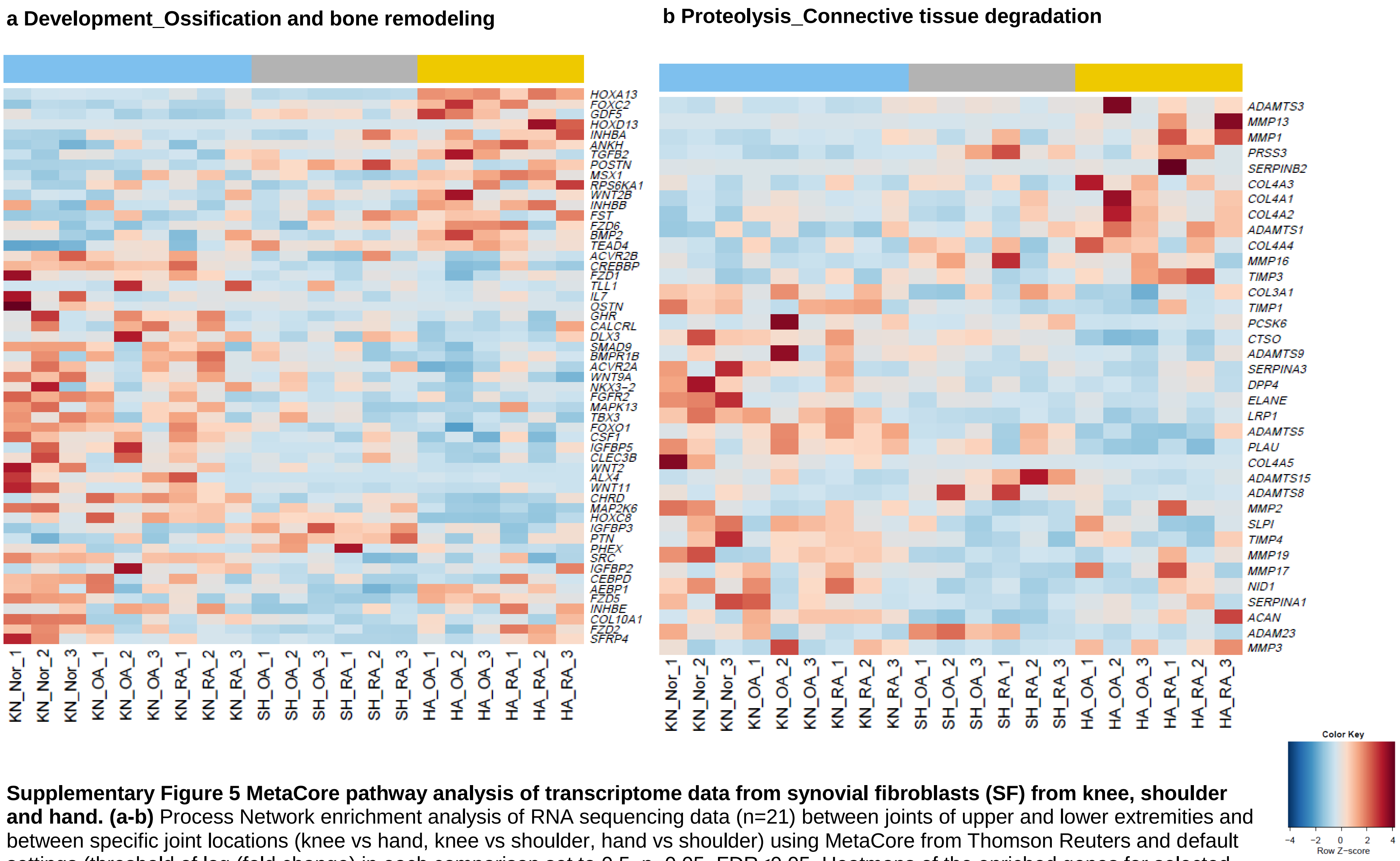

Supplementary Figure 5 MetaCore pathway analysis of transcriptome data from synovial fibroblasts (SF) from knee, shoulder and hand. (a-b) Process Network enrichment analysis of RNA sequencing data (n=21) between joints of upper and lower extremities and between specific joint locations (knee vs hand, knee vs shoulder, hand vs shoulder) using MetaCore from Thomson Reuters and default settings (threshold of log (fold change) in each comparison set to 0.5, p=0.05, FDR<0.05. Heatmaps of the enriched genes for selected arthritis-relevant process networks present among significantly enriched process networks between locations **(a)** Joint-specific network: **"**Development_Ossification and bone remodeling" and **(b)** Arthritis-relevant network: "Proteolysis_Connective tissue degradation»**.**

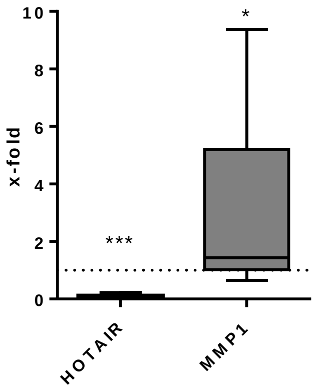

Supplementary Figure 6 Expression of matrix metalloproteinase 1 (*MMP1*) in *HOTAIR*-silenced knee synovial fibroblasts. Silencing of *HOTAIR* in knee OA SF using Antisense LNA™ *HOTAIR* GapmeR (48 h) resulted in increased expression of matrix metalloproteinase 1 (*MMP1*) (n=9, qPCR, data presented as box and whiskers: min to max). X-fold change of Antisense LNA™ *HOTAIR* GapmeR transfected cells over Antisense LNA™ GapmeR Negative Control A transfected cells (set to 1 as depicted by a dotted line). Statistics: two tailed paired t-test, * $p<0.05$; *** $p<0.001$.

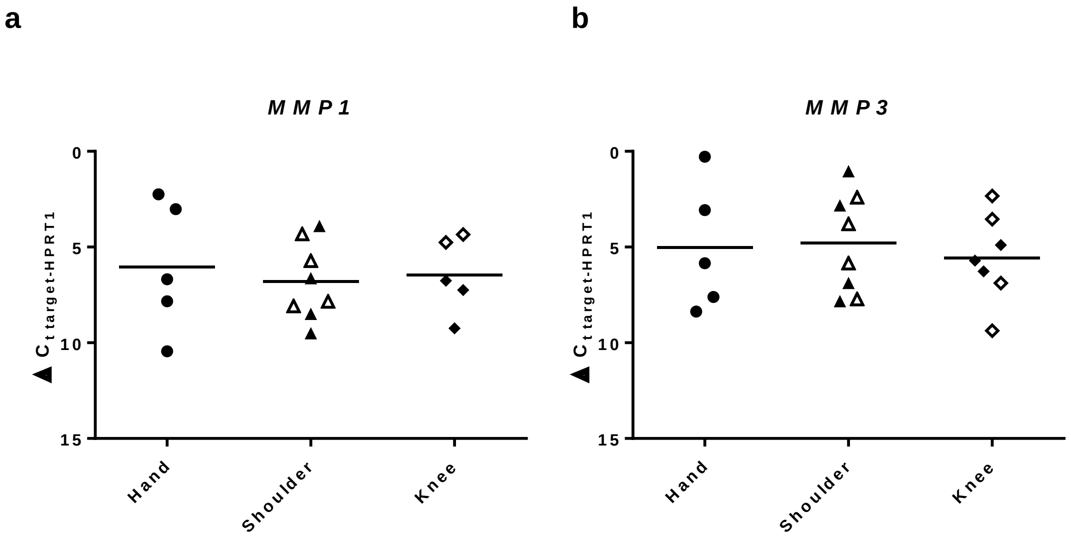

Supplementary Figure 7 Expression of matrix metalloproteinase 1 (*MMP1*) and *MMP3* in synovial tissues from different joints. The expression of **(a)** matrix metalloproteinase 1 (*MMP1*) and **(b)** *MMP3* did not differ between synovial tissues from hand, shoulder and knee joints of RA and OA patients, qPCR. Statistics: 1way ANOVA with Bonferroni’s post test. Open shapes are OA SF, closed shapes are RA SF.

Supplementary Table 1

Characteristics of patients from which synovial fibroblasts were obtained for positional RNA sequencing (n=21)

Joint	Diagnosis	Passage	Sex	Age (years)	Disease duration (years)	Treatment
knee	OA	5	M	64	NA	
knee	OA	5	F	61	NA	
knee	OA	7	F	72	NA	
knee	RA	6	F	54	16	DMARD, biologic, NSAID, corticosteroid
knee	RA	5	F	64	7	DMARD, biologic
knee	RA	6	F	71	51	DMARD, NSAID, corticosteroid
knee	arthralgia	7	F	59		
knee	arthralgia	7	F	49		
knee	arthralgia	7	M	42		
shoulder	OA	6	F	88	NA	
shoulder	OA	7	F	78	NA	
shoulder	OA	7	F	68	NA	
shoulder	RA	6	F	68	38	Biologic, corticosteroid
shoulder	RA	6	F	78	32	Biologic
shoulder	RA	6	F	68	27	DMARD
MCP	OA	6	M	68	NA	
CMC	OA	6	F	61	NA	
CMC	OA	6	F	55	NA	
MCP	RA	6	F	52	21	DMARD
MCP	RA	5	F	69	50	DMARD, NSAID
MCP	RA	6	F	78	23	DMARD, corticosteroid

OA = osteoarthritis, RA = rheumatoid arthritis, F = female, M= male, NA = not assessed; DMARD = disease modifying anti-rheumatic drug; NSAID = non-steroidal anti-inflammatory drug; MCP = metacarpophalangeal joint; CMC = carpometacarpal joint.

Supplementary Table 2

Patient's characteristics for synovial fibroblasts used for measurements of HOX gene expression by qPCR

Joint	Diagnosis	N	Sex	Age (years; min-max)	Disease duration (years; min-max)	Treatment (number of patient receiving drug)
hands (MCP, wrist)	RA	4	F	69 (51-77)	22 (15-50)	NSAID (1), DMARD (4), corticosteroid (1)
hands (CMC)	OA	4	3F/1M	61 (53-74)	NA	
elbow	RA	7	F	56 (40-80)	18 (8-27)	NSAID (4), DMARD (2), corticosteroids (2), biologics (3)
elbow	OA	1	F	52	NA	
shoulder	RA	4	3F/1M	72 (68-78)	32 (17-38)	DMARD (1), corticosteroids (2), biologics (3)
shoulder	OA	4	3F/1M	67 (64-95)	NA	
hip	RA	3	2F/1M	69 (67-73)	12 (2-28)	NSAID (1), DMARD (3), corticosteroids (1)
hip	OA	3	1F/2M	60 (58-68)	NA	
knee	RA	4	2F/2M	72 (61-79)	20 (5-51)	NSAID (1), DMARD (2), corticosteroids (3), biologics (1)
knee	OA	4	2F/2M	66 (61-77)	NA	
knee	arthralgia	3	2F/1M	49 (42-59)		
ankle	RA	5	3F/2M	55 (27-81)	<1	DMARD (1)
ankle	resolving arthritis	2	F	52 (52-52)	NA	

N = number of patients from which synovial fibroblast were obtained per each location; RA = rheumatoid arthritis, OA = osteoarthritis, NA = not assessed; DMARD = disease modifying anti-rheumatic drug; NSAID = non-steroidal anti-inflammatory drug; MCP = metacarpophalangeal joint; CMC = carpometacarpal joint.

Supplementary Table 3

Patient's characteristics for synovial tissues used for qPCR measurements of HOX transcripts

Joint	Diagnosis	N	Sex	Age (years; min-max)	Disease duration (years; min-max)	Treatment
hands (MCP, wrist)	RA	5	F	62 (52-69)	21 (8-50)	NSAID (2), DMARD (2), corticosteroids (1)
elbow	RA	6	4F/2M	54 (50-65)	16 (13-25)	NSAID (2), DMARD (3), corticosteroids (1), biologic (1)
shoulder	RA	4	3F/1M	69 (65-70)	24 (4-48)	NSAID (2), DMARD (1), corticosteroid (1), biologic (1)
shoulder	OA	4	3F/1M	67 (64-75)	NA	
hip	RA	2	F	77 (74-79)	42 (44-44)	NSAID (2)
hip	OA	4	1F/2M	63 (50-69)	NA	
knee	RA	4	2F/2M	63 (40-71)	20 (9-51)	NSAID (1), DMARD (3), corticosteroids (3), biologics (2)
knee	OA	4	2F/2M	67 (64-72)	NA	NSAID (2)
ankle	JRA	1	F	42	26	NSAID (1), DMARD (1)
ankle	RA	2	F	53 (39-66)	14 (9-18)	NSAID (1), DMARD (1), corticosteroid (1)

N=number of tissues per location, RA = rheumatoid arthritis, OA = osteoarthritis; NA= not assessed; F= female, M = male, DMARD = disease modifying anti-rheumatic drug; NSAID = non-steroidal anti-inflammatory drug; MCP = metacarpophalangeal joint

Supplementary Table 4

Characteristics of healthy wild type and arthritic TNF transgenic mice used for isolation of synovial fibroblasts

	Sex	Age (weeks) when sacrificed	Joint of origin of synovial fibroblasts		
			Front paw	Knee	Ankle
**Wt C57BL/6**					
1	F	6			+
2	F	6			+
3	F	6		+	+
4	F	6	+	+	+
5	F	7	+		+
6	F	7	+		+
7	F	6	+	+	+
8	M	6	+	+	+
9	M	8		+	
10	M	8	+	+	
**TghuTNF C57BL/6**					
1	F	6	+		+
2	M	6	+		+
3	F	6	+	+	+
4	F	6		+	+
5	F	7	+		+
6	F	7	+		
7	F	6	+	+	+
8	F	6	+	+	+
9	M	8		+	
10	M	8		+	

Wt = wild type, TghuTNF = TNF transgenic, F = female, M = male

Supplementary Table 5

Patient's characteristics for rheumatoid arthritis synovial tissues used for scoring by Krenn synovitis score

Joint	Diagnosis	N	Sex	Age Mean years (min-max)	Disease duration Mean years (min-max)	Treatment (number of patients receiving treatment)
hands (MCP, PIP)	RA	15	14F/1M	51 (33-64)	16 (5-36)	NSAID (9), DMARD (11), corticosteroid (6), biologics (2), untreated (2)
	JRA	2	F	40 (38-42)	23 (1 NA)	NSAID (2), DMARD (2), corticosteroid (1), biologics
elbow	RA	6	5F/1M	65 (50-75)	38 (28-49)	NSAID (4), DMARD (2), corticosteroids (2), untreated (1)
shoulder	RA	5	2F/3M	64 (51-71)	54 (30-66)	NSAID (3), DMARD (3), corticosteroid (3), biologics (2)
	JIA	2	2M	44 (31-58)	9 (6-11)	NSAID (1), DMARD (2), corticosteroid (1), biologics (1)
hip	RA	4	3F/1M	71 (67-78)	43 (38-55)	NSAID (2), DMARD (3), corticosteroids (3)
	JIA	2	M	33 (32-33)	8 (2-13)	corticosteroids (2)
knee	RA	6	3F/3M	68 (60-76)	45 (30-66) (1 NA)	NSAID (3), DMARD (3), corticosteroids (3)
	JIA	1	M	58	11	NSAID (1), DMARD (1), corticosteroids (1)

N= number of synovial tissues per location; F = female, M = male; NA = not assessed; DMARD = disease modifying anti-rheumatic drug; NSAID = non-steroidal anti-inflammatory drug; MCP = metacarpophalangeal joint; PIP = proximal interphalangeal joint; JIA = juvenile idiopathic arthritis, RA = rheumatoid arthritis, OA = osteoarthritis.

Supplementary Table 6

Sequences of human primers and probes for SybrGreen and TaqMan qPCR measurements

HPRT1	For: 5' ATG GAC AGG ACT GAA CGT CTT G 3'	Rev: 5' GGC TAC AAT GTG ATG GCC TC 3'
B2M	For : 5' TAG TTA AGT GGG ATC GAG ACA TG '3	Rev: 5' TGG AAT TCA TCC AAT CCA AAT GC 3'
MMP1	For: 5' GCT AAC AAA TAC TGG AGG TAT GAT G 3'	Rev:5' ATT TTG GGA TAA CCT GGA TCC ATA G 3'
HOXA9	For: 5' ATC GAT CCC AAT AAC CCA GCA '3	Rev: 5' GAG TGG AGC GCG CAT GAA '3
HOXA10	For: 5' CCC TTC CGA GAG CAG CAA A '3	Rev: 5' GAA TTG CCC AGG GAA TCC TTC T '3
HOXA11	For: 5' GTC TTC CGG CCA CAC TGA G '3	Rev: 5' TGC GTT GGC CAC TGG AG '3
HOXA11-AS	For: 5' TTC TCC TGG GAG GTC CAA CAG '3	Rev: 5' AGA AAT GGA ACT CGG ACT TGG C '3
HOXA13	For: 5' CAG GCG CAG CCT CCC'3	Rev: 5' GAG ACC ACG TCG GGC AG '3
HOXC-AS1	For: 5' GAC CGA GCT TGA AGA AGT GTA GG '3	Rev: 5' AGG AAG ATG GGA TGG GGA CG '3
HOXC-AS3	For: 5' TGC GAG AGA CCA ATG GGA TTT G '3	Rev: 5' ACA ACT TGA CCC CGC TGA CG '3
HOXC4	For: 5' CCA GAG GGT TGG AAA TTA ATG ATC '3	Rev: 5' GGA CTG TGT TCA GGG ATG TAG C
HOXC10	For: 5' TAT CTC CTC TTT CGC TTC GTT ATC C 3'	Rev: 5' CAA TGA AAT CAA GAC GGA GCA GAG 3'
HOXC8	For: 5' ACA CTC ACA ACT CTA ACT ACC TGT C 3'	Rev: 5' GAG TCC GAA CTT TTC ACT GTT TCT C 3'
HOXC-AS2	For: 5' CCA CCC AAG GAA ATT TGC AGT G 3'	Rev: 5' TTA ATT GTA CTT CGG GCT CGT ATT G '3
HOXC11	For: 5' TAT CTG ACT CTC GCT GTG GGA C '3	Rev: 5' AGC AAG ACA TTG TCG CCG AGG '3
HOXC13	For: 5' AAG TCT CCC TTC CCA GAC GTG 3'	Rev: 5' CTG CAC CTT AGT GTA GGG CAC 3'
HOTTIP	For: 5' CAC AAG CAG GTA CCA CAA AGC CA 3'	Rev: 5' TCT CCT TGC TCA GGG ACT CT 3'
AC012531.25	For: 5' CTG GCA AAC TCT CCC AAC TTA G 3'	Rev: 5' CTG ACT ACT CCG AAC TGC TTG 3'
HOTAIR	For: 5' GAT CCG AAA GCT TCC ACA GTG 3'	Rev: 5'AAC TCC CAG GCC TCA GTG C 3'
TNFa	For: 5' TCC AGG CGG TGC TTG TTC 3'	Rev: 5' GCC TGC CAC GAT CAG GAA 3'
MMP13	For: 5\` TCC TAC AAA TCT CGC GGG AAT 3'<br>Probe: 5\` CAT GGA GCT TGC TGC ATT CTC CTT CAG 3\`	Rev: 5\`GCA TTT CTC GGA GCC TCT CA 3\`

Supplementary Table 7

Sequences of mouse primers for SybrGreen qPCR measurements

B2m	For: 5’ CTT CAG CAA GGA CTG GTC TTT C 3’	Rev: 5’ CAT GTC TCG ATC CCA GTA GAC G 3’
Hoxa9	For: 5’ CCA TCG ATC CCA ATA ACC CGG 3’	Rev: 5’ TCC TTC TCC AGT TCC AGC GTC 3’
Hoxa11	For: 5’ AGC GAG AGT TCT TCT TCA GCG 3’	Rev: 5’ ACT TGA CGG TCG GTG AGG TTG 3’
Hoxa11os	For: 5’ AAT TTG GAG ACC CAC CTC AGG G 3’	Rev: 5’ CCA GCC AAC ATG AGT TAC ACC G 3’
Hoxc8	For: 5’ GCG AGC GTG GTG CAA TAT CC 3’	Rev: 5’ GAG CGT GGG GTC TCA TCC AT 3’
Hoxc10	For: 5’ ATC AAG ACA GAG CAA AGC CTG G 3’	Rev: 5’ GCC TTT ATC TCC TCT TTA GCT TCG 3’
Hottip	For: 5’ GAG GTG CCT TCT CCC TTC TTT G 3’	Rev: 5’ GGA ATC AGA AGC AGG TAC AAC GG 3’
Hotair	For: 5’ CGA CGC CTT CCT TAT AAG CTC 3’	Rev: 5’ CTC TCT CTG CCT TTA CAG TTG C 3’

Supplementary Table 8

Control regions to assess specificity of immunoprecipitation reactions used for Chromatin Immunoprecipitation DNA sequencing

Mark	Positive region	Negative region
**H3K4me3**	*GAPDH* TSS, *EIF4A2* promoter	*MB* exon 2, *Sat2*
**H3K27me3**	*MYT1*, *TSH2B*	*GAPDH* TSS, *EIF4A2* promoter
**H3K4me1**	*ACTB3*, *GAS2L1*	*GAPDH* TSS, *EIF4A2* promoter
**H3K27ac**	*ACTB promoter*, *EIF4A2* promoter	*MYT1*, *TSH2B*
